# Ambient carbon monoxide exposure and elevated risk of mortality in the glioblastoma patients: A double‐cohort retrospective observational study

**DOI:** 10.1002/cam4.3572

**Published:** 2020-11-07

**Authors:** Seon‐Jin Yoon, Juhwan Noh, Hye Young Son, Ju Hyung Moon, Eui‐Hyun Kim, Sahng Wook Park, Se Hoon Kim, Jong Hee Chang, Yong‐Min Huh, Seok‐Gu Kang

**Affiliations:** ^1^ Department of Biochemistry and Molecular Biology Yonsei University College of Medicine Seoul Korea; ^2^ Brain Korea 21 Plus Project for Medical Science Yonsei University College of Medicine Seoul Korea; ^3^ Department of Preventive Medicine Yonsei University College of Medicine Seoul Korea; ^4^ Severance Biomedical Science Institute Yonsei University College of Medicine Seoul Korea; ^5^ Department of Neurosurgery Brain Tumor Center Severance Hospital Yonsei University College of Medicine Seoul Korea; ^6^ Department of Pathology Yonsei University College of Medicine Seoul Korea; ^7^ Department of Radiology Severance Hospital Yonsei University College of Medicine Seoul Korea; ^8^ YUHS‐KRIBB Medical Convergence Research Institute Seoul Korea; ^9^ Department of Medical Science Yonsei University Graduate School Seoul Korea

**Keywords:** ambient air pollution, carbon monoxide, glioblastoma, mortality

## Abstract

An increasing number of studies indicate air pollutants infiltrate into the brain. We aimed to find the association of cumulative air pollution exposure in the main body of primary brain tumor: glioblastoma (GBM). In this double‐cohort, retrospective analysis study with a protocol, we compared the health effect of air pollution on the GBM patients from the SEER (Surveillance, Epidemiology, and End Results Program) in 27 U.S. counties from 10 states and GBM patients of Severance cohort of Korea. From 2000 to 2015, 10621 GBM patients of the SEER were individually evaluated for the cumulative average exposure for each pollutant, and 9444 (88.9%) mortality events were reported. From 2011 to 2018, 398 GBM patients of the Severance with the same protocol showed 259 (65.1%) mortality events. The multi‐pollutant models show that the association level of risk with CO is increased in the SEER (HR 1.252; 95% CI 1.141‐1.373) with an increasing linear trend of relative death rate in the spline curve. The Severance GBM data showed such a statistically significant result of the health impact of CO on GBM patients. The overall survival gain of the less exposure group against CO was 2 and 3 months in the two cohorts. Perioperative exposure to CO may increase the risk of shorter survival of GBM patients of the SEER and the Severance cohort.

## Graphical abstract

This retrospective observational study found that chronic exposure to ambient level of carbon monoxide is associated with a shorter survival of glioblastoma patients (2‐3 months). Such association was validated in the cohorts of the United states (N=10621, HR 1.252, 95% CI 1.141‐1.373) and Korea (N=398, HR 2.874, 95% CI 1.040‐7.944).

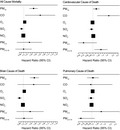

## 1 INTRODUCTION

Accumulation of evidence shows the air pollutants infiltrate into the brain, changes the genetic (or epigenetic) status of DNA, or may deteriorate oligodendrocytes.^1^, ^2^, ^3^, ^4^ Thus, we hypothesized that the mortality of brain tumor patients might be associated with air pollution.^5^

We focused on the glioblastoma (GBM) patients who are classified as the most vulnerable group among the brain tumor patients.^6^ GBM is one of the devastating primary tumors of the brain with the median survival about 11 to 30 months.^6^, ^7^, ^8^ These patients suffer from the rapid progression of the disease, and the patients are prone to stay in the hospital after the surgical operation.^9^ And after they are discharged from the hospital, the trip of the patient is limited by the severity, emotional distress, brain dysfunction, or comorbidity from the disease.^10^

World health organization (WHO) provide a general guideline on the exposure limit on the particulate matters (PM_10_ and PM_2.5_), carbon monoxide (CO), ozone (O_3_), nitric oxide (NO_2_), and sulfur oxide (SO_2_) for the general population with the potential health effect.^11^, ^12^ However, it is still uncertain whether these general recommendations can be utilized for the risk stratification of brain tumor patients.

We aimed to find whether an exposure level within a specific time‐window to ambient air pollution is associated with the clinical course of GBM. We applied the result of the exploratory step to the protocol‐based retrospective observational analysis of two cohorts: the Severance cohort (Korea) and the SEER (Surveillance, Epidemiology, and End Results Program, United States) database of GBM to verify our hypothesis that whether the mortality of GBM is associated with specific air pollutant.

## 2 MATERIAL AND METHODS

### 2.1 Study design and data sources

In this protocol‐based retrospective observational study, we analyzed the GBM cohort of the Severance hospital and the SEER cohort (Table 1, Supporting information Section A). Air pollutants (PM_10_, PM_2.5_, PM_2.5‐10_, O_3_, CO, SO_2_, and NO_2_) data were used for calculation of the individual cumulative exposure for the specified time‐window.^13^, ^14^ According to the recommendation of the institutional review board and the SEER data use agreement; we anonymized and analyzed the gathered data to protect the privacy of patients. We excluded the patients from the analysis if more than 30% of exposure data is missing for each pollutant. In line with the WHO criteria of the recommended exposure for the air pollutants, we created the cumulative average exposure models for this study and the perioperative exposure model based on the exploratory step (Supporting information Figure C1). The global minimum of the Euclidean distance between each patient and the closest local air pollution monitoring station was used to allocate patients to the nearest station. All the analysis included in this study includes patients within the 10 km (six miles) distance from the monitoring station to the residential area of the patients after the distance analysis (Supporting information Figure D1–6, Figure E6–10). Exposure details and further analysis for each cohort are provided in the appendix.

**Table 1:** The clinical characteristics of patients

	Severance cohort	SEER cohort
Patients	(n = 398)	(n = 10 621)
Mortality events	259	9444
Mean exposure		
PM_10_ (µg/m^3^)	48.5 ± 14.7	25.6 ± 11.1
CO (ppm)	0.5 ± 0.1	0.5 ± 0.3
Age	56.7 ± 13.5	64.0 ± 13.9
Sex		
Male	242 (60.8%)	6 255 (58.9%)
Female	156 (39.2%)	4 366 (41.1%)
Race		
Asian	398 (100%)	838 (7.9%)
White	0 (0%)	7 390 (69.6%)
Hispanic	0 (0%)	1 789 (16.8%)
Black	0 (0%)	571 (5.3%)
Others	0 (0%)	33 (0.3%)
Surgery		
Yes	398 (100%)	7 632 (71.9%)
No	0 (0%)	2 989 (28.1%)
Chemotherapy		
Yes	325 (81.6%)	5 655 (53.2%)
No	73 (18.3%)	4 966 (46.7%)
Radiation mode		
Adjuvant radiation	335 (84.2%)	5 592 (52.6%)
No	63 (15.8%)	5 029 (47.4%)
Radiation		
Beam radiation	361 (90.7%)	6 970 (65.6%)
No	37 (9.3%)	3 651 (34.4%)

Data are n (%) and mean with standard deviation. Diagnosis of all patients is made as glioblastoma.

PM_10_: Particulate matter with an aerodynamic diameter less than 10 µm, CO: Carbon monoxide, ppm: Parts per million.

### 2.2 The protocol‐based procedures

Single‐pollutant model analysis of comparing the Severance cohort and the SEER cohort was performed (Table 2). Primary analysis harmonized the criteria of selecting patients by the distance from the monitoring stations and the adjusting variables between the different cohorts. The perioperative exposure was estimated by the 1‐month exposure of the month that the diagnosis of GBM was made in the hospital or the month of surgical operation. Subgroup analysis by the molecular markers was performed in the Severance cohort, as neither IDH (Isocitrate dehydrogenase) mutation status and MGMT (O‐6‐methylguanine‐DNA methyltransferase) promoter methylation status were available in the SEER cohort (Table 2). Multi‐pollutant models included the same adjusting factors as the single‐pollutant models (Table 3). The sensitivity tests were done excluding one factor from the multivariate‐adjusted models (Supporting information Figure E1–5). The cause‐of‐death analysis and other susceptibility tests were studied in the SEER cohort.

**Table 2:** The hazard ratio of single‐pollutant models with the increment of a single unit.

Models	Severance Model 1^a^	*p*	Severance Model 2^b^	*p*	Severance Model 3^c^	*p*	SEER Model	*p*
Exposure	N = 398		N = 361		N = 37		N = 10 621	
PM_10_ (10 µg/m^3^)	1.078 (0.994–1.171)		1.095 (1.007–1.192)	**0.034**	0.887 (0.586–1.341)		1.044 (1.025–1.063)	**<0.0001**
CO (1 ppm)	3.034 (1.483–6.206)	**0.0023**	2.745 (1.336–5.642)	**0.0059**	91.225 (1.032–8066.238)	**0.048**	1.075 (1.006–1.148)	**0.031**
Ozone (1 ppb)	0.994 (0.982–1.006)		0.996 (0.984–1.009)		0.99 (0.933–1.05)		1.004 (1.002–1.006)	**<0.001**
SO_2_ (1 ppb)	1.09 (1.026–1.159)	**0.0057**	1.09 (1.024–1.159)	**0.0066**	1.06 (0.685–1.64)		0.990 (0.983–0.998)	**0.014**
NO_2_ (1 ppb)	1.01 (0.999–1.021)		1.011 (0.999–1.022)		0.992 (0.941–1.046)		0.998 (0.996–0.999)	**0.032**

All models are adjusted by age, sex, radiation method, radiation surgery sequence, chemotherapy status, and race. Bold indicates significant values (*p* < 0.05).

PM: particulate matter, CO: carbon monoxide, O_3_: ozone, SO_2_: sulfur dioxide, NO_2_: nitrogen dioxide, ppm: parts per million, ppb: parts per billion.

^a^ All GBM patients included for the Cox model.

^b^ IDH‐wild‐type GBM model.

^c^ IDH‐mutant GBM model.

**Table 3:** Hazard ratio of multi‐pollutant models with the increment of a single unit

	Severance Cohort	*p*	SEER Cohort	*p*
Exposure	N = 398		N = 10 621	
PM_10_ (10 µg/m^3^)	0.994 (0.896 –1.103)		1.004 (1.002–1.006)	**<** **0.0005**
CO (1 ppm)	2.874 (1.040 –7.944)	**0.041**	1.252 (1.141–1.373)	**<0.0001**
Ozone (1 ppb)	1.006 (0.99–1.022)		1.003 (1.000–1.005)	**0.023**
SO_2_ (1 ppb)	1.065 (0.985–1.151)		0.991 (0.983–0.999)	**0.032**
NO_2_ (1 ppb)	1 (0.984–1.017)		0.994 (0.991–0.996)	**<** **0.0001**

The models are adjusted by all the included pollutant levels, age, sex, race, radiation method, radiation surgery sequence, chemotherapy status, and race. Bold indicates significant values (*p* < 0.05).

PM: Particulate matter, CO: Carbon monoxide, O_3_: Ozone, SO_2_: Sulfur dioxide, NO_2_: Nitrogen dioxide, ppm: Parts per million, ppb: Parts per billion.

### 2.3 Primary outcomes

We used the overall survival mortality as the primary outcome in the analysis. We gathered the mortality data of Severance cohort from the cancer registry of Severance hospital, which records the survival of oncology patients from death certificates, national health insurance survival data, and electronic medical records. The SEER database provided the mortality data and the cause of deaths with the registration and acceptance of the agreement form.

### 2.4 Statistical analysis

Cox proportional‐hazards regression was used to estimate hazard ratios and 95% confidence intervals (CIs) for the time to the first mortality event in the GBM patients associated with an elevation of 10 μg per cubic meter in the level of cumulative average exposure to particulate matters (PM_10_, PM_2.5_, and PM_2.5‐10_) for both cohorts. Other pollutants were assessed with the harmonized units between the cohorts (ppm for CO; ppb for O_3_, SO_2_, and NO_2_). To estimate the concentration‐response association of exposure and the relative death rate, we fit a penalized spline curve (degree of freedom = 4). Cyclic patterns of air pollutants were calculated using the autocorrelation method.

## 3 RESULTS

### 3.1 The air pollutants of two cohorts.

**Figure 1:**
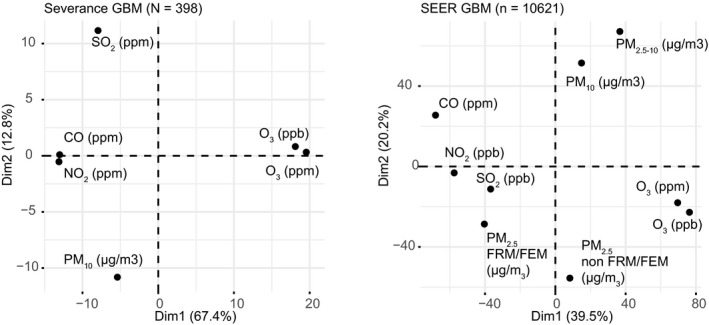
PCA plot of air pollutants in the protocol‐based analysis. The individual exposure data of GBM patients for the cohort of the Severance and the SEER database. PCA: The principal component analysis, Dim: Dimension, GBM: Glioblastoma, ppm: Parts per million, ppb: Parts per billion, PM: Particulate matter, FRM/FEM: A federal reference method and a federal equivalent method of measuring PM_2.5_, CO: Carbon monoxide, SO_2_: Sulfur dioxide, NO_2_: Nitric dioxide, O_3_: Ozone.

We evaluated the baseline characteristics of air pollution over the years of Korea and the United States. Overall, the level of PM_10_ decreased in recent years (Supporting information Section Figure B.1–4). The PM_10_ shows a seasonal cyclic pattern with a peak in different seasons. CO shows a decreasing trend in Korea and the United States with cyclic seasonal pattern (Supporting information Section Figure B.5–6). The dimensional reduction technique revealed PM_10_, O_3,_ and CO are relatively independent in the two databases (Figure 1), allowing us to compare the health impact of these pollutants.

### 3.2 The discovery step of the Severance cohort

Before the primary analysis, we examined the discovery step to find the time‐window from the date of operation whether the cumulative average of specific air pollutants affect the survival of the GBM patients (Supporting information A.4). We implemented long‐term and short‐term time‐windows and compared the results in the preoperative (in the residential address of patients) and the postoperative exposure window (in the hospital). We made Cox hazard models that adjust for age, sex, and molecular markers (IDH and MGMT promoter methylation) and visualized the results with differential time‐windows (Supporting information Figure C3–10). The elevated exposure level to PM_10_, CO, and sulfur dioxide was associated with the poor overall survival of the GBM patients (Supporting information Table C1). The preoperative exposure shows the difference of median survival with PM_10_ and CO (Supporting information Figure C2).

From this finding, we hypothesized that the surrogate estimate of the 1‐month perioperative exposure might replicate the risk elevation pattern in the Severance cohort. Thus, the method can be applied for the SEER database (Supporting information Figure C1).

### 3.3 The demographics of the two cohorts

The characteristics of the two cohorts for the primary analysis share the diagnosis of GBM, age (older than 19 years), and the distance range within the 10 km (six miles) from the air monitoring stations (Table 1). The mean age of diagnosis is younger in the Severance cohort (56.7 vs 64.0). Male patients consisted of two‐third of the patients in both groups (60.8% vs 58.9%). For both cohorts, the pollutant models were adjusted by age, sex, surgical sequence (relative to radiotherapy), type of radiation, the status of chemotherapy, and race (different races were included and adjusted in the SEER model, not in the Severance models). The diagnosis of all patients of Severance cohort was from the surgical confirmation of the pathologic slides (100%), and the diagnostic methods of the SEER include surgical diagnosis (9778, 91.8%), clinical diagnosis (747, 7.0%), and other methods (116, 1.0%). Further details of the cohort are described in Table D1,2 (Severance) and Table E1,2 (SEER) of Supporting information.

### 3.4 Protocol‐based retrospective analysis with the perioperative exposure model

We built a protocol that can validate our hypothesis in the Severance cohort and the SEER cohort (Supporting information A.10). Individual‐level exposure was estimated for each pollutant in the perioperative period (1‐month exposure when the diagnosis was made). As the molecular marker IDH mutation status was available for Severance GBM patients, we compared the risk of air pollution effect on all 398 patients considering the marker, while not in the SEER cohort which lacks the molecular marker information (Table 2).

### 3.5 Elevated mortality risk by the CO and PM_10_ exposure

In the single‐pollutant models, CO was the only one air pollutant statistically significant (Table 2). The level of hazard ratio was attenuated in the SEER cohort (HR 1.075; 95% CI 1.006‐1.148) than the Severance cohort (HR 3.034; 95% CI 1.483‐6.206). Elevated risk by the CO remained and more accentuated in the IDH‐mutant patients (HR 91.225; 95% CI 1.032‐8066.238, 9.6% of total GBM patients).

**Figure 2:**
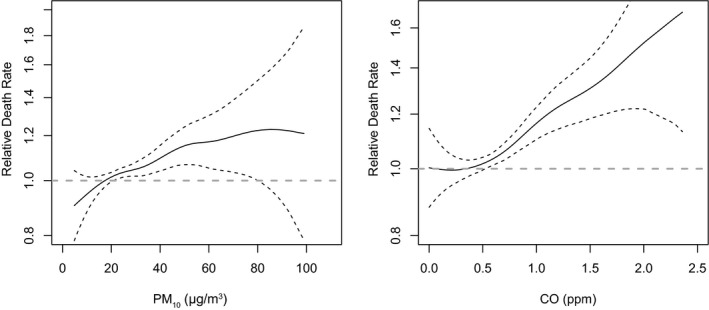
Therelative death rate of the multi‐pollutant models in the SEER cohort. The estimates of both graphs were adjusted for age, sex, race, radiotherapy type, surgery to radiation sequence, and the status of chemotherapy. PM_10_: particulate matter with an aerodynamic diameter less than 10 µm, CO: carbon monoxide, ppm: parts per million.

PM_10_ was found to be associated with the elevated risk in the subgroup of Severance cohort (HR 1.095; 95% CI 1.007‐1.192, Table 2) with IDH‐wild‐type GBM which comprises 90.3% of GBM as well as in the SEER cohort (HR 1.044; 95% CI 1.025‐1.063, Table 2). The association of health risk from the ambient CO was also found in the multi‐pollutant model (Table 3). And the multi‐pollutant models showed a dose‐response effect with the relative death rate with CO (Figure 2).

The health effect of PM_10_, O_3_, and SO_2_ were not consistent over the two different cohorts (Table 2 and 3). While the SEER cohort shows the elevated risk from O_3_ in the GBM (HR 1.004; 95% CI 1.002‐1.006), the Severance cohort does not show such a result (Table 2). SO_2_ was associated with the elevated in the all GBM of Severance cohort (HR 1.09; 95% CI 1.026‐1.159) and not consistent in the SEER cohort (Table 2).

From the primary analysis of the perioperative exposure model, we selected PM_10_ and CO as the possible risk associated air pollutant in the GBM. We assessed the association in a larger data set of Severance cohort that includes non‐GBM samples in the same study period (Supporting information Figure D1). The Kaplan–Meier curve of GBM shows the median survival of PM_10_ was not significantly different, and that of CO was significant (PM_10_, 16 months for both groups, *p* = 0.16; CO, 15 vs 18 months, Log‐rank *p* = 0.011). The survival of non‐GBM was not associated with the above pollutants.

### 3.6 Cause of death analysis in the SEER cohort

**Figure 3:**
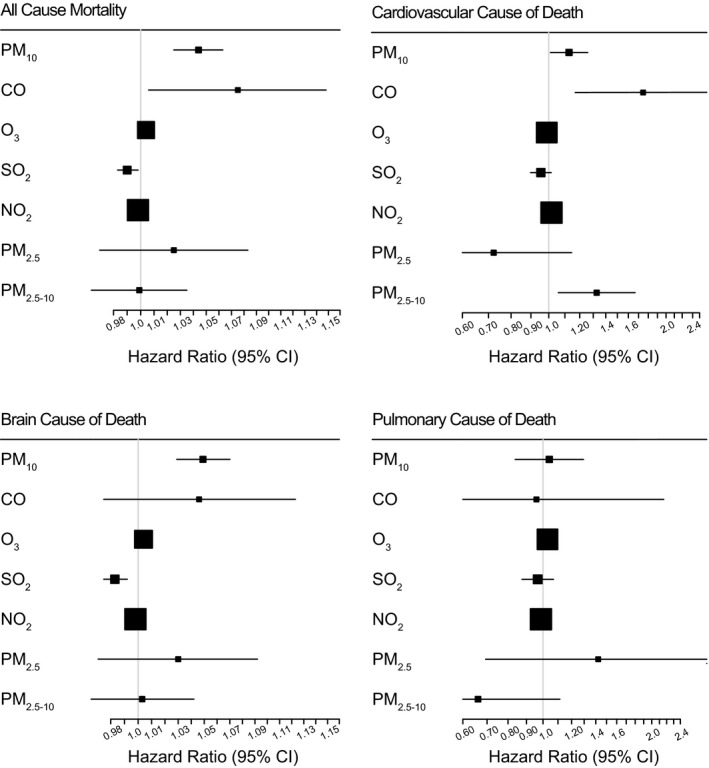
Thesingle‐pollutant models and the cause of death in the SEER database. These models were adjusted by age, sex, race, surgical sequence to radiation, radiation type, and chemotherapy status. PM: Particulate matter, CO: Carbon monoxide, O_3_: Ozone, SO_2_: Sulfur dioxide, NO_2_: Nitrogen dioxide.

We also did a cause‐of‐death analysis in the SEER cohort (Figure 3). PM_10_ showed associations with the overall cause, brain cause, and cardiovascular cause mortality (that includes the cerebrovascular cause death) of GBM. CO showed associations with the overall and the cardiovascular cause mortality (that includes cerebrovascular cause death). Ozone showed overall, brain, and pulmonary cause mortality, which may be consistent with the prior result.^15^ Additionally, PM_2.5‐10_ was associated with an elevated risk in the cardiovascular mortality of GBM (Figure 3).^2^, ^16^ More details are provided in Table E3 of Supporting Information.

### 3.7 Sensitivity and susceptibility analysis

The sensitivity test of the SEER shows that the statistical significance of PM_10_ and O_3_ remained stable with the exclusion of a variable from the main full‐adjusted model (Supporting information Figure E1,E3). The models of CO and SO_2_ revealed that the associations vary by the sensitivity test (Supporting information Figure E2,E4).

The susceptibility shows the elevated risk of PM_10_ and CO remained stable in the by sex, radiation‐surgery sequence, and chemotherapy (Supporting information Table E4). Older patients were more vulnerable to exposure to CO, and younger patients were associated with PM_10_. Patients with beam radiation were not associated with the elevated risk of CO exposure (HR 1.047; 95% CI 0.966‐1.136).

We also assessed other factors, such as the location of patients, the year of diagnosis, and the month of diagnosis. Inequal population among states was found in the subgroup analysis: 73% of patients were from the California state, and the number of patients from nine states were without association with these two air pollutants when combined as a group (Supporting information Table E5). When the patients are spread to 16 years, there was no association in the CO with the elevated risk of mortality, while PM_10_ shows an intermittent association (Supporting information Table E6). The month of diagnosis also shows no consistent pattern of association with PM_10_ and CO (Supporting information Table E7).

### 3.8 Difference in the median survival

In the discovery step, the estimated survival benefits of PM_10_ and CO were three months each (Supporting information Figure C2). The protocol‐based Severance perioperative exposure model shows the statistical significance remained by the exposure level against CO with three months of survival gain in the lower exposure group of GBM (Supporting information Figure D1). The SEER data show that the estimated benefit of the low exposure group of PM_10_ and CO shows two months of survival gain in the lower exposure group (Supporting information Figure E12).

## 4 DISCUSSION

Carbon monoxide (CO) is a continuing problem for the global society.^17^, ^18^, ^19^ In addition to the previously known acute high‐level exposures,^17^ our study revealed that chronic ambient‐level exposure against CO is associated with a significantly deteriorated survival of patients. In this retrospective double‐cohort observational study, we validated the perioperative exposure models of individual patients of GBM, and we associated the risk of overall survival with the level of exposure. We found that ambient‐level CO showed the most significant adverse health effect, suggesting a chronic low‐level exposure can shorten the survival of GBM patients. While the impact of CO in the SEER cohort was relatively attenuated than the Severance cohort, the hazard ratio of cardiovascular death shows more elevated risk (Figure 3) as well as in the multi‐pollutant model (Table 3). The brain tumor mortality was reported as not affected by the air pollution in the Cancer Prevention Study‐II.^20^, ^21^ However, we found the first association of CO with the survival of GBM patients in the double‐cohort setting.

The air pollutant, CO, may be interpreted as the factor that influences the survival of the patient.^22^ it is still uncertain whether the air pollutant affected the nonbiological factors, the baseline patient condition, other organs such as heart, other factor/cells in the bloodstream, cancer‐origin‐related cell (such as oligodendrocyte progenitor cells [OPC]), the tumor cell itself, the necrotic portion of the disease, or its surrounding cells.^23^, ^24^ We speculate that if CO can deteriorate OPCs,^3^ the delayed neurological sequelae‐associated effects of CO can be exaggerated or accelerated in the OPC‐related GBM.^25^, ^26^

The limitations include the characteristics of this study, methodology, included patients, behavioral differences, and migration issues. As this is an observational study, our result shows a preliminary example of the association of the air pollutant and not address the causality of the phenomenon. The cumulative average method is a well‐known method with known limitations in the field of industrial epidemiology and pharmaceutical epidemiology.^14^, ^27^, ^28^ To manage the issue of long‐term exposure, we assessed the extended timeframes of differential windows up to nearly 1800 days prior to the surgery, and we found that the seasonal variation may bias the results (Supporting information Section B or Figure C3–5). The spatial resolution of 10 km (six miles) also restricted the population of GBM of both cohorts (Supporting information Figure D2–6). Especially, the SEER cohort from 42 303 to 10 621 for the limited number of air monitoring station with 73% of the population in California (Supporting information Figure E6‐E10). The small number of patients that does not include all population of GBM of Korea and the United States (Based on the U.S. population in 2015, about 15% of GBM were included for this study), and with the most patients from California (Supporting information Table E5). As we did not consider the humidity and temperature of each region for the method we applied, the confounding effect of climate was not examined in this study. Considering the seasonal pattern of Korea and California, the winter season might be associated with the mortality pattern. The behavioral difference between Korean and U.S. patients should be considered when interpreting the results.^9^, ^29^ Due to Korean national health insurance coverage, there is an overall difference in the patient behavior of the length of stay in the hospital after the brain tumor operation: Korean stays longer than the U.S. patients (19 days vs 3‐6 days).^9^, ^30^ Migration rate should have less affected the outcome of this study than other long‐term studies.^21^, ^22^ The lower migration rate of the census data shows that about 95% of patients lived in the same county in the last year in the United States (Supporting information Table E1,E2). And we should mention that there are other unknown factors inherent in our study that should have influenced our results.

In the design process for this study with STROBE guidelines, we hypothesized that the perioperative exposure could be used to show the difference in the survival in the patients based on the discovery data (Supporting information A.10). Our first aim of finding a pollutant was achieved with CO from both cohorts and not with PM_10_ in the Severance cohort. Our second aim of quantifying the health effect on the overall survival was achieved: Even the two databases of Severance and the SEER show a relatively robust risk pattern of CO, the difference of median survival by the exposure level is three months in the Severance cohort and two months in the SEER database (Supporting information Figure E12). This double‐cohort, retrospective observational result of an association from the air pollutants found a major subset of brain tumor patients who are more vulnerable to chronic CO exposure.^20^, ^21^

In summary, we found that the elevated exposure level to CO is associated with the elevated risk of mortality of GBM patients in this double‐cohort retrospective study. Even the survival gain is 2 to 3 months, considering the devastating short survival of glioblastoma and recent findings on the adverse health effect on the brain, this association warrants further biological study.

## CONFLICT OF INTERESTS

All the authors declare no competing interests.

## AUTHOR CONTRIBUTIONS

S‐JY, Y‐MH, and S‐GK conceived, designed the study. S‐JY, JHM, E‐HK, SHK, JHC, and S‐GK collected samples and constructed the database for the research. S‐JY performed the statistical analysis and wrote the first draft of the manuscript. S‐JY, JN, HYS, SWP, and Y‐MH critically reviewed the protocols and supervised the study. S‐JY, Y‐MH, SWP, and S‐GK revised the manuscript. All authors had full access to the study data, discussed and reviewed the manuscript, and approved the manuscript for publication.

## ETHICAL DECLARATIONS

The institutional review board approved this study at Severance Hospital, Yonsei University, the Republic of Korea before commencing the overall study: Severance cohort of the Republic of Korea (IRB 4‐2018‐1221) and the SEER cohort of the United States (IRB‐4‐2019‐0960).

## Supporting information

Supplementary Material

## Data Availability

The air pollution data that support the findings of this study is openly available in EPA homepage at https://aqs.epa.gov/aqsweb/airdata/download_files.html#Raw. The cancer registry data of the United States is available in the SEER homepage at https://seer.cancer.gov/ with a data usage request.
