# Supplementary material for: Ambient carbon monoxide exposure and elevated risk of mortality in the glioblastoma patients: A double‐cohort retrospective observational study
Source: Cancer Med. 2020 Nov 7;9(23):9018–26. doi: 10.1002/cam4.3572 (PMC7724304; doi:10.1002/cam4.3572)
Supplement: Supplementary file 1 — Supplementary Material [file CAM4-9-9018-s001.pdf]

Supporting Information

Ambient carbon monoxide exposure and elevated risk of mortality in the glioblastoma patients: A double-cohort retrospective observational study

Table of contents

SECTION A: Extended methods and the protocol 4

APPENDIX TEXT 4

A.1 Ethical declarations 4

A.2 Severance study subjects 4

A.3 Severance patient exposure against air pollutants 4

A.4 The differential time-window of discovery step. 5

A.5 Mortality Outcomes of Severance Cohort 5

A.6 SEER study subjects 5

A.7 SEER study cohort assignment to monitoring stations 6

A.8 Mortality Outcomes of SEER Cohort 6

A.9 Statistical methods 6

A.10 Protocol for the main analysis 7

SECTION B: Overall air pollutants in Korea and the United States. 11

APPENDIX FIGURES 11

Fig. B.1 Overall Trend of Particulate Matter$_{10}$ in Seoul, Republic of Korea. 11

Fig. B.2 Postoperative Exposure Window of PM_{10} of Severance Cohort. 12

Fig. B.3 Pattern of Particulate Matter$_{10}$ in a Monitoring Station of California 13

Fig. B.4 Pattern of Particulate Matter$_{10}$ in a Monitoring Station of Iowa 14

Fig. B.5 Carbon monoxide in Korea 15

Fig. B.6 Carbon monoxide in California, the United states 16

SECTION C: Discovery step 17

APPENDIX TABLES 17

Table C.1 Discovery phase Cox models in the Severance cohort. 17

APPENDIX FIGURES 18

Fig. C.1 Overall Workflow 18

Fig. C.2 Overall survival in the discovery step of the Severance cohort. 19

Fig. C.3 Long-term Preoperative Exposure Window of PM_{10} of Severance Cohort. 20

Fig. C.4 Long-Term Preoperative Exposure of Severance Cohort of GBM. 21

Fig. C.5 Long-Term Postoperative Exposure of Severance Cohort of GBM. 22

Fig. C.6 Short-Term Preoperative Exposure Window of PM_{10} and carbon monoxide of Severance Cohort. 23

Fig. C.7 Preoperative cumulative average Cox hazard model of the Severance GBM cohort. 24

Fig. C.8 Postoperative cumulative average Cox hazard model of the Severance GBM cohort. 25

Fig. C.9 Short-Term Preoperative Exposure of Severance Cohort of GBM. 26

Fig. C.10 Short-Term Postoperative Exposure of Severance Cohort of GBM. 27

SECTION D: Protocol-based validation in the Severance cohort. 28

APPENDIX TABLES 28
Table D.1 Demographics of Severance cohort study subjects and events, according to the exposure groups against PM_{10} (N=398). 28
Table D.2 Demographics of Severance cohort study subjects and events, according to the exposure groups against CO (N=396). 29
APPENDIX FIGURES 30
Fig. D.1 Survival curve of the perioperative exposure of the Severance cohort. 30
Fig. D.2 Distance Analysis of PM_{10} in the Severance Cohort. 31
Fig. D.3 Distance Analysis of CO in the Severance Cohort. 32
Fig. D.4 Distance Analysis of O_3 in the Severance Cohort. 33
Fig. D.5 Distance Analysis of SO_2 in the Severance Cohort. 34
Fig. D.6 Distance Analysis of NO_2 in the Severance Cohort. 35
SECTION E: Protocol-based validation in the SEER cohort. 36
APPENDIX TABLES 36
Table E.1 Demographics of SEER GBM patients by quintile of PM_{10} (N=10 621). 36
Table E.2 Demographics of SEER GBM patients by the level of CO (N=10 621). 38
Table E.3 Causes of death of the SEER GBM patients in the perioperative single-pollutant models. 39
Table E.4 Cox hazard model of single-pollutant by the subgroup of GBM in the SEER cohort. 40
Table E.5 Cox hazard model of single-pollutant by the location in the SEER cohort. 41
Table E.6 Cox hazard model of single-pollutant by the year of diagnosis in the SEER cohort. 42
Table E.7 Cox hazard model of single-pollutant by the month of diagnosis in the SEER cohort. 43
APPENDIX FIGURES 44
Fig. E.1 Sensitivity Analysis of PM_{10} in the SEER Cohort. 44
Fig. E.2 Sensitivity Analysis of CO in the SEER Cohort. 45
Fig. E.3 Sensitivity Analysis of O_3 in the SEER Cohort. 46
Fig. E.4 Sensitivity Analysis of SO_2 in the SEER Cohort. 47
Fig. E.5 Sensitivity Analysis of NO_2 in the SEER Cohort. 48
Fig. E.6 Distance Analysis of PM_{10} in the SEER Cohort. 49
Fig. E.7 Distance Analysis of CO in the SEER Cohort. 50
Fig. E.8 Distance Analysis of O_3 in the SEER Cohort. 51
Fig. E.9 Distance Analysis of SO_2 in the SEER Cohort. 52
Fig. E.10 Distance Analysis of NO_2 in the SEER Cohort. 53
Fig. E.11 Distribution of perioperative exposure to patients in the SEER cohort. 54
Fig. E.12 The survival curve of the SEER database. 55
APPENDIX REFERENCES 56

SECTION A: Extended methods and the protocol

APPENDIX TEXT

A.1 Ethical declarations

The institutional review board approved this study at Severance Hospital, Yonsei University, Republic of Korea before commencing overall study: Severance cohort of the Republic of Korea (IRB 4-2018-1221), and the SEER cohort of the United States (IRB-4-2019-0960).

A.2 Severance study subjects

We collected GBM patient data from the clinical data repository system of Severance Hospital (Seoul, Republic of Korea) from 2011 to 2018. We restricted our study population to the pathologically confirmed GBM with Isocitrate dehydrogenase (IDH) mutation status available samples (n = 422).[1-3] We included adult patients (20 or older) and excluded childhood gliomas (younger than 20 at the diagnosis of the disease).

A.3 Severance patient exposure against air pollutants

We geocoded residential area (or zip-code level address) of each patient as longitude and latitude to estimate the level of exposure to air pollutants. We converted the old version of the patient address (Land name address) to the newer version (Road name address) using the publicly available Korean government website (www.juso.go.kr). We excluded if the patients came from a foreign country if they are living outside of the country without any Korean address. The global minimum of the Euclidean distance between each patient and the closest local air measurement station (Korean Government) was used to allocate patients to the nearest station. We excluded the patients if they are located more than 6.213 miles (or 10 kilometres) away from the station. We visualized the location of patients and the measuring stations with the ggmap package 2.6.1 in R, ggplot2 package 3.1.1 in R.

We processed the data of the Korean air monitoring station (www.airkorea.or.kr, 2018) for the Severance cohort. This database contains the nationwide hourly air pollution data (PM_{10}, SO_2, NO_2, CO, and O_3 level) measured from 2000 to 2018. We calculated the 8-hour run average of ozone to harmonize the results with that of the SEER database with

17 observation per day. The exposure data is calculated from the closest local measuring station from the residential address of the patients.

A.4 The differential time-window of discovery step.

The preoperative long-term exposure group includes the cumulative average data of 1 to 1,831 days before the first operation date for brain cancer with an interval of 30 days (62-time points).

Preoperative short-term exposure includes the cumulative average data of 1 to 51 days with an interval of 10 days (6-time points).

The postoperative exposure was measured in the nearest measuring station from the Severance hospital, and the cumulative exposure was calculated with 1 to 37 days with a two-day interval (19-time points).

A.5 Mortality Outcomes of Severance Cohort

We gathered the mortality data from the cancer registry of Severance hospital, which records the survival of oncology patients from death certificates, national health insurance survival data, and electronic medical record (EMR).

A.6 SEER study subjects

GBM patient data for primary analysis were gathered from the 2017 Submission of the Surveillance Epidemiology and End Results (SEER) Program of the National Cancer Institute. The database collected information on all GBM diagnosed among residents of 13 U.S. states and 604 counties (42 321 patients, from 2000 to 2015).[4] Louisiana cases are included except the hurricane Katrina period from July 2005 to December 2005. If the patients are diagnosed more than once for GBM, only the first record is preserved for the analyses. Information evaluated from SEER included: patient age, sex, race, county, states, the month of diagnosis, surgery-radiation sequence, radiation type, chemotherapy status, and follow-up vital status. According to the International Classification of Diseases for Oncology 3rd edition, glioblastoma, NOS (9440/3), giant cell glioblastoma (9441/3), and gliosarcoma (9442/3) were included as the main

target diseases. Overall survival was the primary outcome. Causes of deaths were also examined in the subgroup analysis.

A.7 SEER study cohort assignment to monitoring stations

Ambient levels of PM_{10} were estimated by the Euclidean assignments to the nearest monitoring station from the residential area of patients. Other air pollutants are analyzed in the method similar to PM_{10}. Air pollution data were retrieved from Environmental Protection Agency (EPA) from 2000 to 2015 (in which 1,040 monitoring stations for PM_{10} in 2000, 355 monitoring stations for PM_{10} in 2015). We applied the quality control process (QC process) by the distance and quality of the air pollution data for each patient. The QC process includes the distance limit of six miles (10 km) from the air monitoring station to the centroid of the residential area of the SEER database, and the measurement more than 70% of the specified period. Which lead to 10 U.S. states and 30 counties (10 621 patients). The perioperative monthly average of daily arithmetic mean value was assigned to individual patients at the monitoring station level. Patient data missing more than 30 % of data were excluded from the study. We used the unmodified data of PM_{10} (81102), $PM_{2.5}$ FRM/FEM (88101), ozone (44201), carbon monoxide (42101), sulfur dioxide (42401), nitric dioxide (42602) for the all analysis in this study.[5] $PM_{2.5-10}$ were calculated by the PM_{10} and $PM_{2.5}$ values from EPA.

A.8 Mortality Outcomes of SEER Cohort

Mortality data and cause of death were included in the case-listing data of the SEER database. We divided cause of death into five groups, brain-specific cause, cardiovascular cause, cardiovascular causes without cerebrovascular causes, pulmonary system causes, and gastrointestinal system causes. Brain-specific causes do not include other causes of deaths. Cardiovascular causes include hypertension, heart disease, atherosclerosis, aortic aneurysm or dissection, other arteriole diseases and cerebrovascular diseases. Pulmonary system causes include chronic obstructive pulmonary disease, pneumonia, or other lung diseases.

A.9 Statistical methods

Exposures level (y_i) of the pollutants (i) were assigned to all subjects which is different by the month and the year of diagnosis of GBM.

$$\lambda_{\text{single-pollutnant (i)}}(t) = \lambda_0(t) \cdot e^{(\beta y_i + \gamma k_i)} \qquad \text{Eq. (A.1)}$$

Model (1) estimated the overall single-pollutant effect of PM_{10}, $PM_{2.5}$, $PM_{2.5\text{-}10}$, O_3, CO, SO_2, and NO_2 which are adjusted by the clinical covariates.

$$\lambda_{\text{multi-pollutants}}(t) = \lambda_0(t) \cdot e^{\left(\beta y_{i1} + \beta y_{i2} + \beta y_{i3} + \beta y_{i4} + \beta y_{i5} + \gamma k_{\text{all pollutants}}\right)} \qquad \text{Eq. (A.2)}$$

Model (2) estimated the overall multi-pollutant effect of the respected pollutants. We excluded the coarse ($PM_{2.5\text{-}10}$ and the fine particulate matter ($PM_{2.5}$) from the adjusting factors.

Stratified Cox proportional hazards regression was performed to estimate hazard ratios (HR) and 95% confidence intervals (CI) for time to first mortality event associated with a unit difference for each pollutant.

A.10 Protocol for the main analysis

A.10.1 Specific aims

Aim 1

To assess the relationship between the exposure to each air pollutant and clinical outcomes of GBM patients. We hypothesized that the perioperative exposure history of air pollutants is associated with significant differences in clinical outcomes of overall survival of GBM patients in the independent cohorts.

Aim 2

To quantify the health effect of particular air pollutant on the overall survival of GBM patients.

A.10.2 Study design

This study is a multi-cohort, retrospective study. Data will be collected from March 1, 2018, to March 1, 2019, from the Severance hospital and the SEER cohort. A schematic of the design appears in appendix p 8.

A.10.3 Sample size

We estimate our sample size calculation based on prior works which reported the impact of air pollutants on the cancer patients and the exploratory step of our study.[6,7] We conservatively estimated the relative hazard of PM_{10} exposed group over the less exposed group as 1.09 following the exploratory phase. For 80% power and an α of 0.05, we will need a total sample size 4227 events (or 2114 per group). We also estimated the doubled relative hazard of CO exposed group over the less exposed group in the exploratory step. For 80% power and an α of 0.05, we will need a total sample size 65 events (or 33 per group).[8] With two databases have a difference in the characteristics (table 1), we decided to analyze the data separately. We expect a total sample size that can achieve the objectives of this investigation in the SEER cohort.

A.10.4 Anticipated results

We anticipate that the level of exposure to particulate matter, carbon monoxide, and sulfur dioxide is associated with the overall survival of GBM patients with the perioperative exposure model of the Severance cohort. We also anticipate that the air pollutants are associated with the elevated risk of death in the SEER cohort.

A.10.5 Data storage and management

All data will be entered by the principal investigators (PI) or research assistants, and data accuracy will be verified by the study PI. Data quality control measures will include queries to identify missing data with quality control data, distance from the air monitoring stations, outliers, removal of duplicate patient data, and discrepancies. Only research assistants and site PIs will have access to protected health information. A unique identifier will be assigned to each study subject. The data from all sites will be downloaded and stored using a password-protected research computer. All computers will be password protected and encrypted. The PI will ensure that the anonymity is maintained. Patients will not be identified by name in any reports on this study, and the result will be presented with the statistical table and figure formats. The study PI will have access to the final study dataset.

A.10.6 Ethics and dissemination

To enhance reporting quality and transparency, this study will be reported in accordance with the Strengthening the Reporting of Observational Studies in Epidemiology Statement: Guidelines for Reporting Observational Studies (STROBE).

Data and resources will be shared with other eligible investigators through academically established means. The datasets used or analyzed during the study will be available from the corresponding author on reasonable request. Collaboration with other investigators interested in the application of the calculation method of air pollutant exposure to other cancers will be welcomed. The results from this work will be published as a full-length, peer-reviewed manuscript.

A.10.7 Strengths and limitations

Strengths

Currently, two retrospective cohort study has been published about the potential health effect of the air pollutant in the brain tumour patients. This study will address and bridge the gap of some of the prior knowledge in this field: (1) the multi-cohort nature of the study will enhance the validity of the findings and (2) cumulative exposure with the well-controlled variables. This study will allow us to find an association of the health effect of the air pollution, by doing so, healthcare professionals and policymakers to consider the health effect of the air pollutants, biological researchers to find therapeutic vulnerabilities of specific cancer.

Limitations

This study will have several limitations. As an observational study, it will only be able to show associations and not causation. We will try to increase causal inference in several ways. We will apply rigorous multivariable analysis and subgroup analysis to address potential confounders. The consistency of our results will be checked with prior work in this field and the data from the different sources. Finally, we will report all results transparently in accordance with STROBE guideline. The observational cohort study design is also prone to confounders, though this should be reduced somewhat by prospective data collection. The collection of exposure data with the specified one-month period would be advantageous for the association between the air pollutant exposure and the length of

survival, as we already conducted the differential time-window assessment with the discovery cohort of the Severance Hospital. Finally, the intent of this observational study is not to produce definitive answers related to the health effect air pollution on the GBM patients, but rather to provide further exploratory data in this area and more preliminary data for a more extensive amount of data. In that regard, this multi-cohort study could stimulate other researchers to find the vulnerable groups within the previously neglected group of cancer patients or other weaker population groups.

SECTION B: Overall air pollutants in Korea and the United States.

APPENDIX FIGURES

Fig. B.1 Overall Trend of Particulate Matter$_{10}$ in Seoul, Republic of Korea.

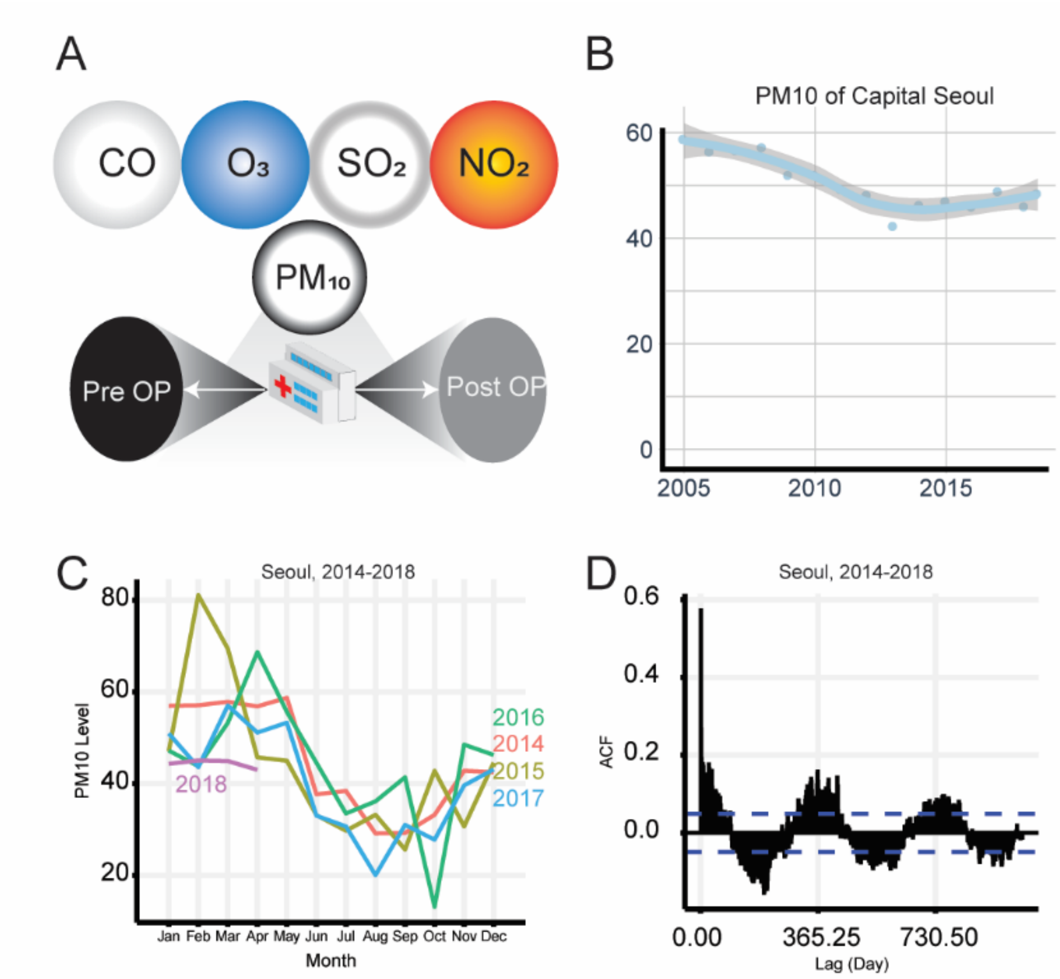

(A) The design of this study including the five air pollutants, and particulate matter (particles with a mass median aerodynamic diameter of less than 10 μm [PM$_{10}$]) was considered more extensively in the main analysis. (B) The mean concentration of PM$_{10}$ in the central area of Seoul, Republic of Korea (Jung-gu). (C) The seasonal plot of PM$_{10}$ from 2014 to 2018. (D) The result of autocorrelation of PM$_{10}$ from 2014 to 2018.

Fig. B.2 Postoperative Exposure Window of PM_{10} of Severance Cohort.

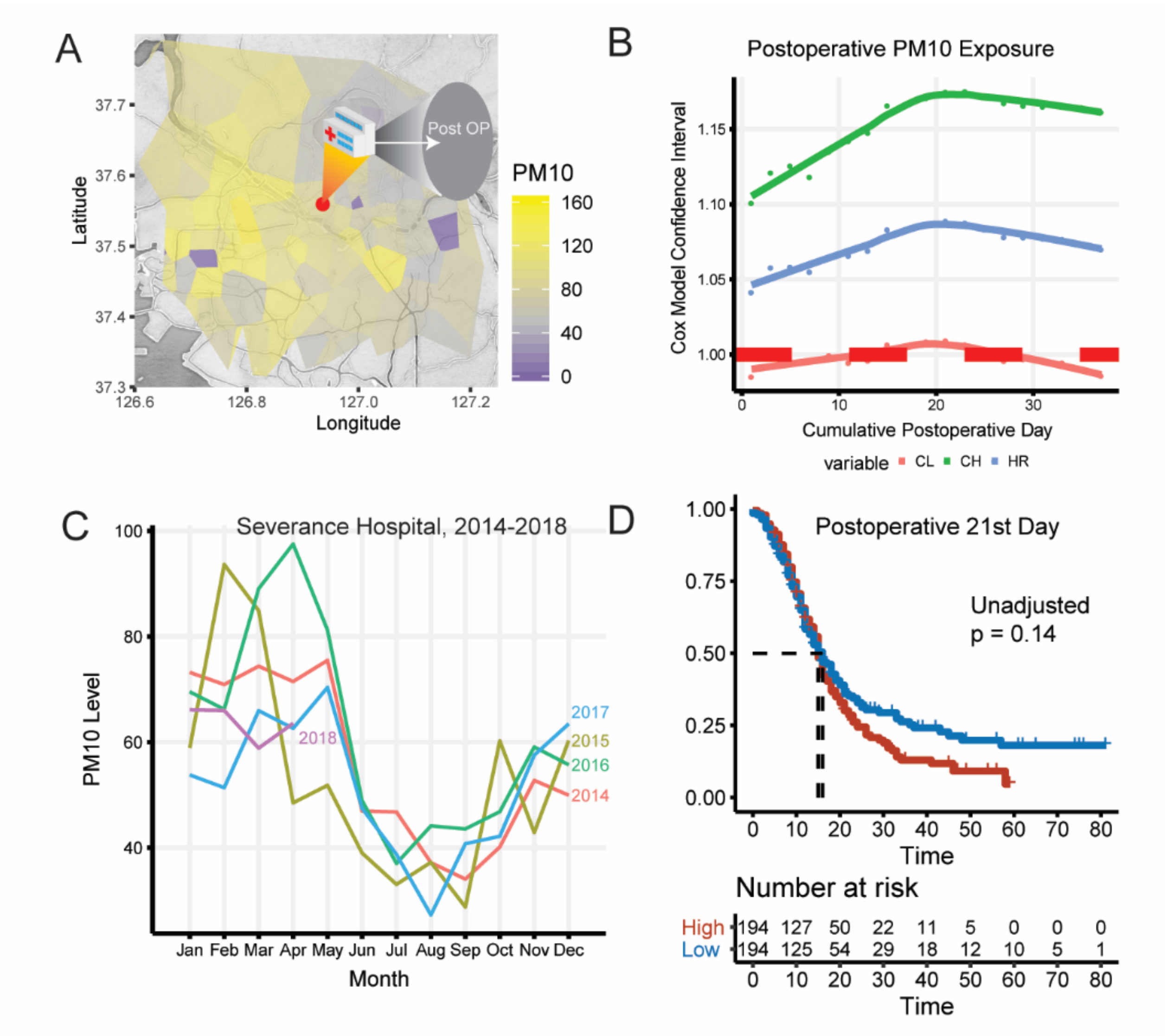

(A) The map shows the PM_{10} concentration of a time point of April 2016. **(B)** the 95 % confidence interval of the single-pollutant Cox hazard ratio. **(C)** Seasonal plot of the level of PM_{10} ($\mu g/m^3$) of the monitoring station near the Severance hospital. **(D)** the Kaplan-Meier curve of the postoperative 21st day of GBM.

Note: Cox model was adjusted by the age, sex, IDH mutation status, and MGMT methylation status of short-term postoperative exposure window (Blue indicates the hazard ratio of the model, Red indicates lower limit of the interval, Green indicates the upper limit of the confidence interval)

Fig. B.3 Pattern of Particulate Matter$_{10}$ in a Monitoring Station of California

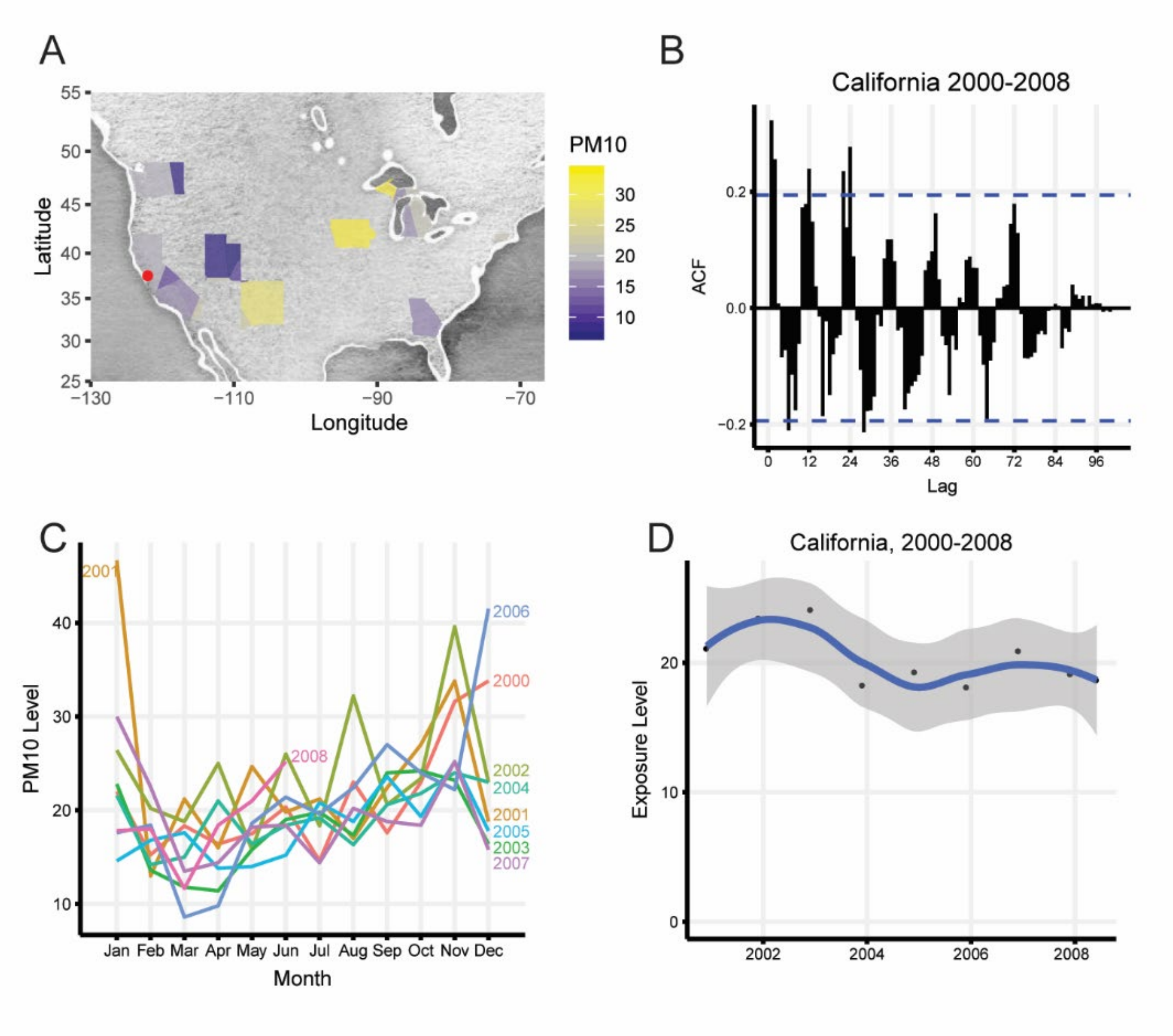

(A) The air pollutant level on the SEER registry with red dot represents an air pollutant monitoring station of September 2012. (B) The autocorrelation result of PM$_{10}$ a California monitoring station (Lag, month). (C) The seasonal plot of the level PM$_{10}$ (μg/m^3) of the monitoring station of California. (D) The mean daily concentration of PM$_{10}$ (μg/m^3) averaged by year in a California measuring station from 2000 to 2008.

Fig. B.4 Pattern of Particulate $Matter_{10}$ in a Monitoring Station of Iowa

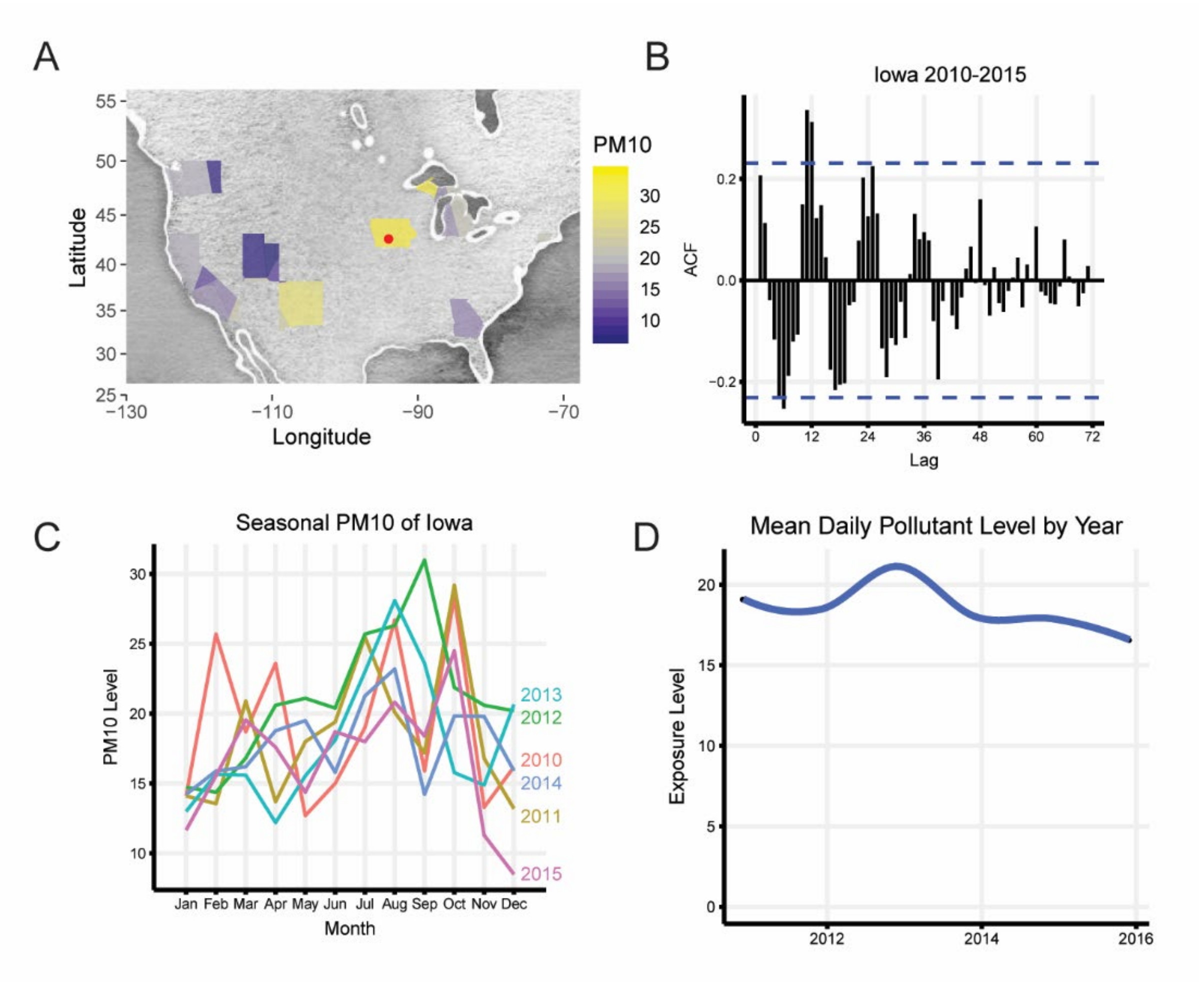

(A) The air pollutant level on the SEER registry. Map shows the PM_{10} concentration of September 2012. Red dot represents a measuring station of Iowa state used in these figures. (B) The autocorrelation result of PM_{10} the Iowa measuring station (Lag, month). (C) The seasonal plot of PM_{10} of the monitoring station of Iowa. (D) The mean daily concentration of PM_{10} averaged by year in the central area of Iowa measuring station from 2010 to 2015.

Fig. B.5 Carbon monoxide in Korea

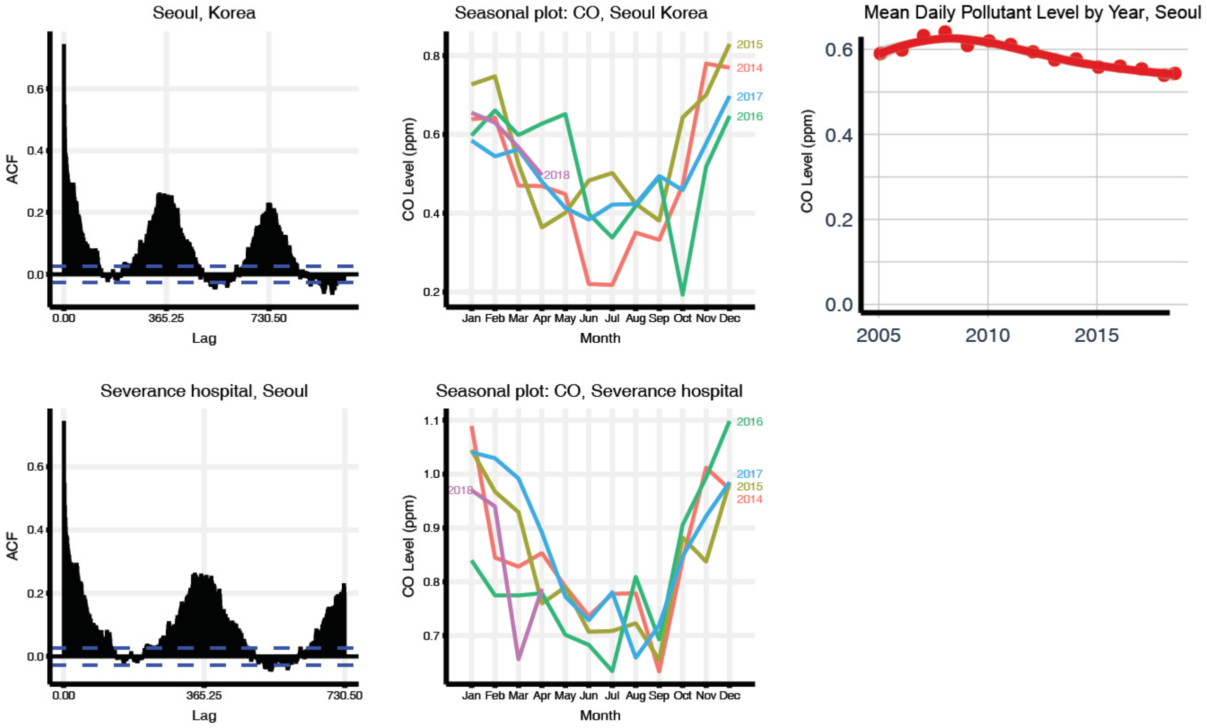

Comparison of ambient CO level between Seoul (Upper row) and Severance hospital (Lower row). **ACF:** Autocorrelation function (Lag = 365 days). **CO:** Carbon monoxide

Fig. B.6 Carbon monoxide in California, the United states

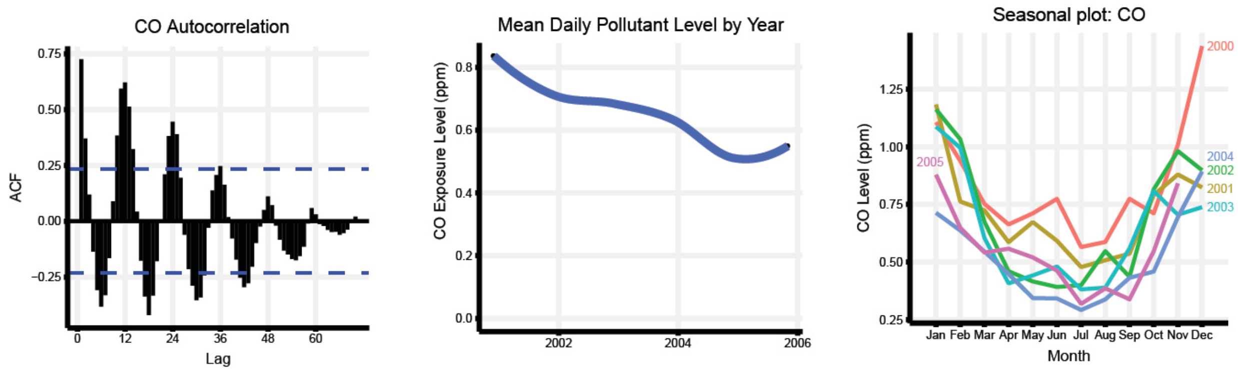

An example of the monitoring results of San Francisco-Oakland-Hayward.

ACF: Autocorrelation Function, **CO:** Carbon monoxide

SECTION C: Discovery step

APPENDIX TABLES

Table C.1 Discovery phase Cox models in the Severance cohort.

Air pollutants	Unit	Preoperative model [a]	Postoperative model [b]
PM_{10}	10 μg/m^3	**1.096 (1.007 - 1.193)**	**1.088 (1.008 - 1.174)**
Carbon Monoxide	1 ppm	**2.027 (1.070 - 3.840)**	1.828 (0.926 - 3.607)
Ozone	1 ppb	0.991 (0.979 - 1.003)	0.994 (0.983 - 1.006)
SO_2	1 ppb	**1.070 (1.005 - 1.139)**	**1.060 (1.002 - 1.121)**
NO_2	1 ppb	1.008 (0.998 - 1.019)	1.004 (0.994 - 1.014)

[a] The result of the cumulative average from the preoperative 31st day to the operation date.
[b] The cumulative average of the exposure against each pollutant from the operation date to the 21st postoperative day.

APPENDIX FIGURES

Fig. C.1 Overall Workflow

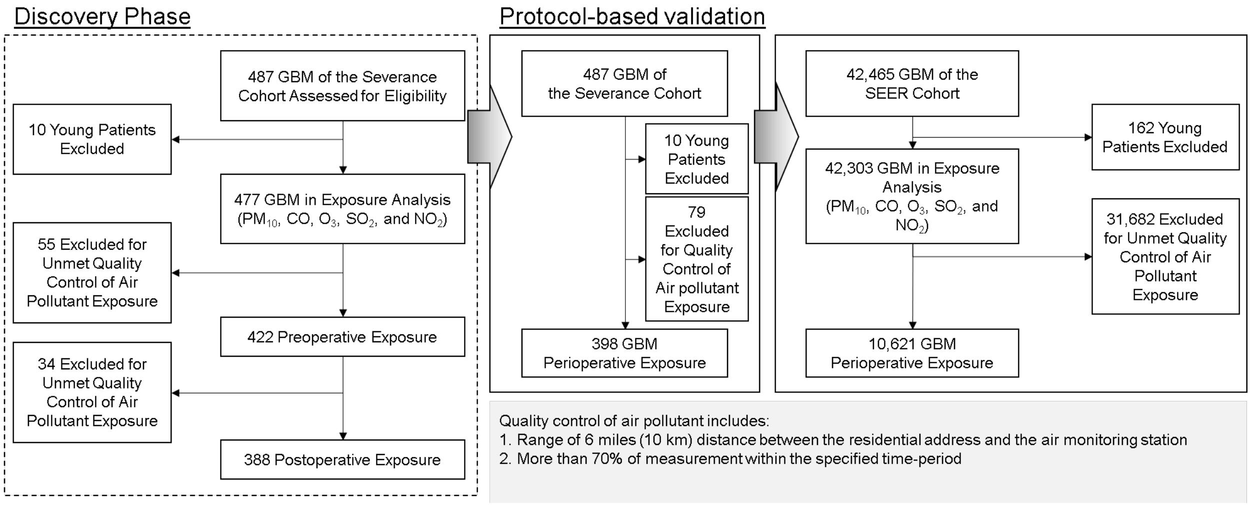

Discovery step is adjusted by age, sex, IDH mutation status, and MGMT methylation status of the GBM patients. Protocol-based validation is adjusted by age, sex, race, chemotherapy status, radiation-surgery sequence, and radiation type.

Fig. C.2 Overall survival in the discovery step of the Severance cohort.

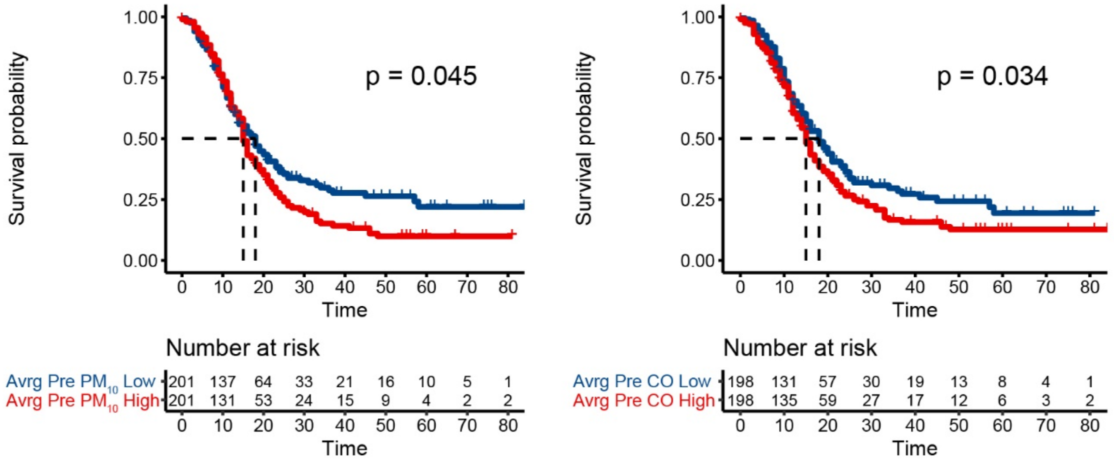

PM= particulate matter, CO = carbon monoxide, Avrg Pre = cumulative average of exposure of 11 days before the surgical operation of GBM. Both pollutants distinguished the median survival of GBM as 15 months vs 18 months.

Fig. C.3 Long-term Preoperative Exposure Window of PM_{10} of Severance Cohort.

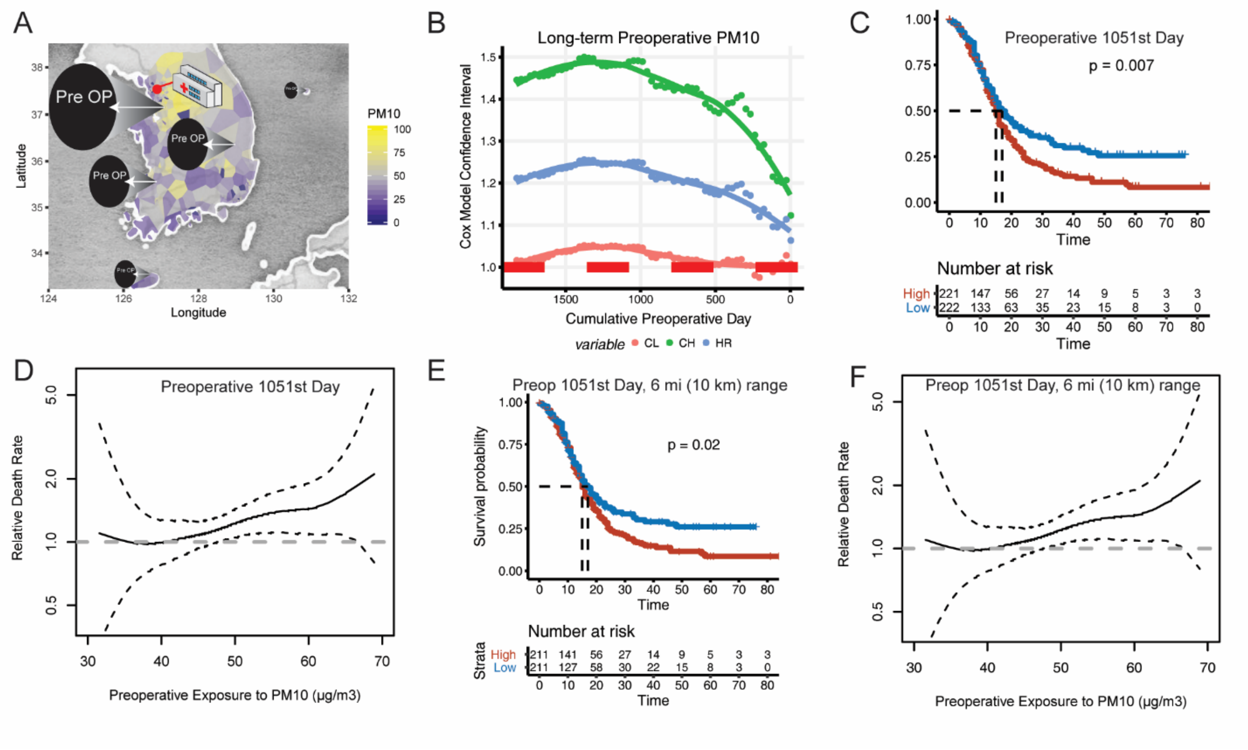

Panel A shows the design of preoperative exposure window for the GBM patients. Map shows the PM_{10} concentration in the Saturday afternoon of March 2018. Panel B shows the 95 % confidence interval of the single-pollutant Cox hazard model adjusted by the age, sex, IDH mutation status, and MGMT methylation status. Panel C shows the Kaplan-Meier curve of overall survival in the preoperative 1051st day of GBM patients (Median survival 15 vs 17 months). Panel D shows the spline curve of the single pollutant model of cumulative average from 1051st preoperative day to the operation day which is adjusted by the age, sex, IDH mutation, and MGMT methylation status. Panel E shows the Kaplan-Meier curve of overall survival within the 6 mi (10km) range from the air monitoring stations (Median survival 15 vs 17 months). Panel F shows the spline curve within the 6 mi (10km) distance from the air monitoring stations.

Fig. C.4 Long-Term Preoperative Exposure of Severance Cohort of GBM.

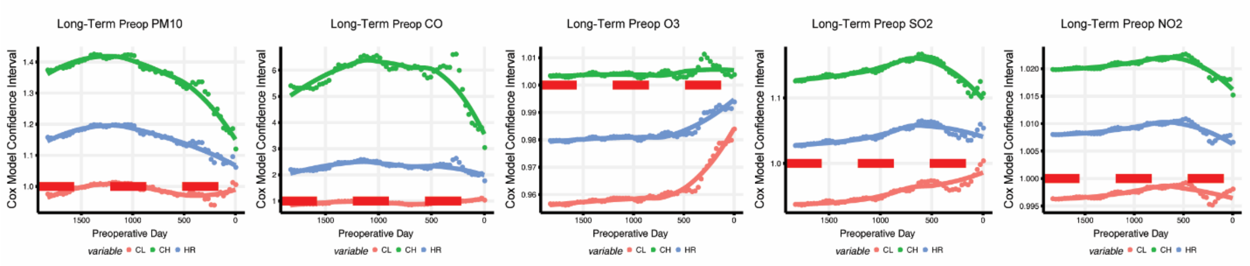

Preoperative single pollutant Cox Hazard models of cumulative average of PM_{10}, CO, O_3, SO_2, and NO_2 are adjusted by age, sex, the IDH mutation status, the MGMT methylation status from preoperative 1831 days to the 1 day.

Fig. C.5 Long-Term Postoperative Exposure of Severance Cohort of GBM.

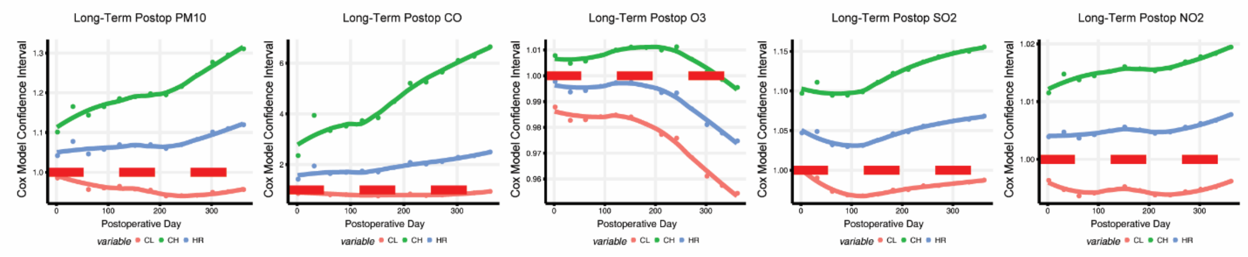

Postoperative single pollutant Cox Hazard models of cumulative average of PM_{10}, CO, O_3, SO_2, and NO_2 are adjusted by age, sex, the IDH mutation status, the MGMT methylation status from postoperative 331st day to the 1st day.

Fig. C.6 Short-Term Preoperative Exposure Window of PM_{10} and carbon monoxide of Severance Cohort.

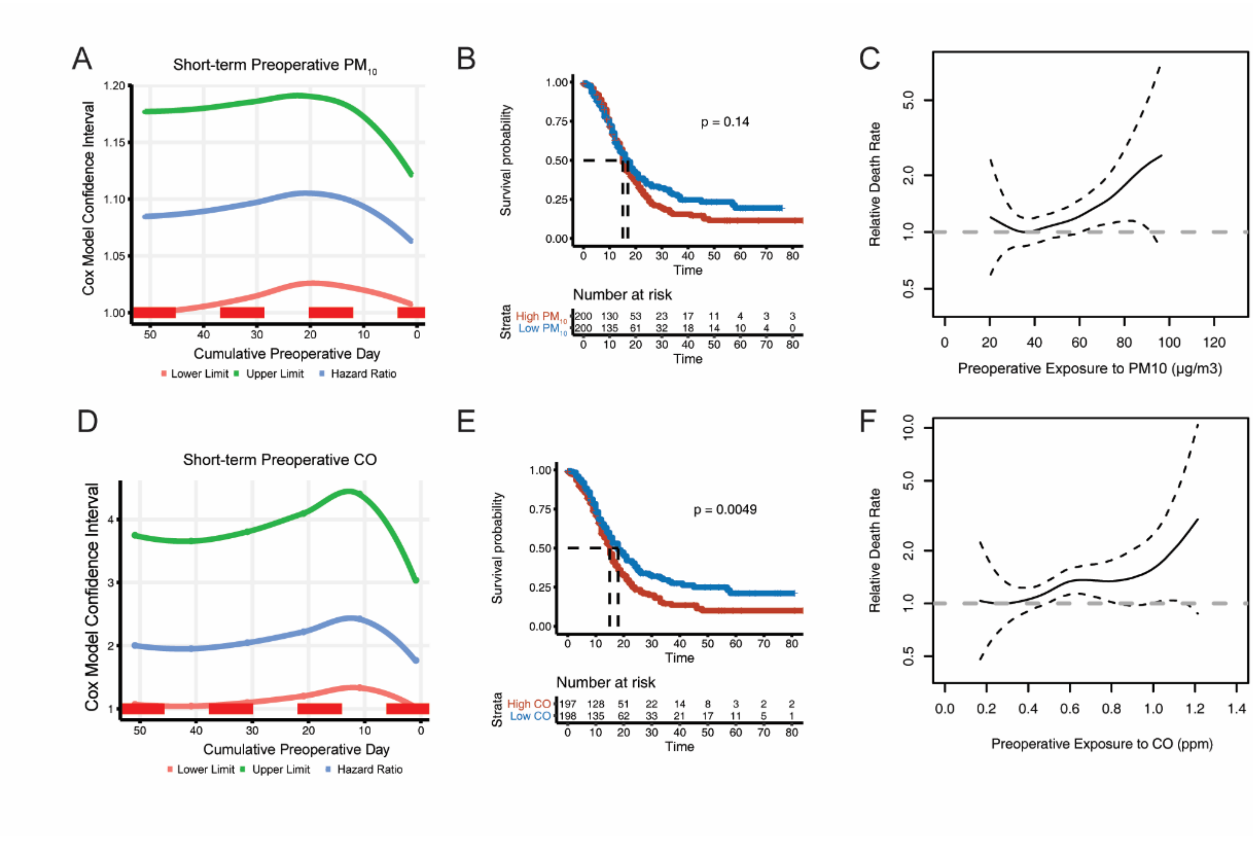

(A, B, C) Short-term preoperative PM_{10} and the survival of GBM. **(A)** The 95% confidence interval of the single-pollutant Cox hazard model. **(B)** Overall survival by the level of PM_{10} (Median survival 15 vs 18 months) **(C)** The spline curve of PM_{10}. **(D, E. F)** Carbon monoxide and the survival of GBM. **(D)** the single-pollutant Cox hazard model **(E)** By carbon monoxide (Median survival 15 vs 18 months). **(F)** The spline curve with carbon monoxide.

Note: KM plots and spline curves are depicted with the preoperative 31 cumulative day average of each pollutant. Cox models and spline curve are adjusted by age, sex, IDH mutation, and MGMT methylation status. (**A, D**: Blue indicates the hazard ratio of the model, Red indicates lower limit of the interval, Green indicates the upper limit of the confidence interval)

Fig. C.7 Preoperative cumulative average Cox hazard model of the Severance GBM cohort.

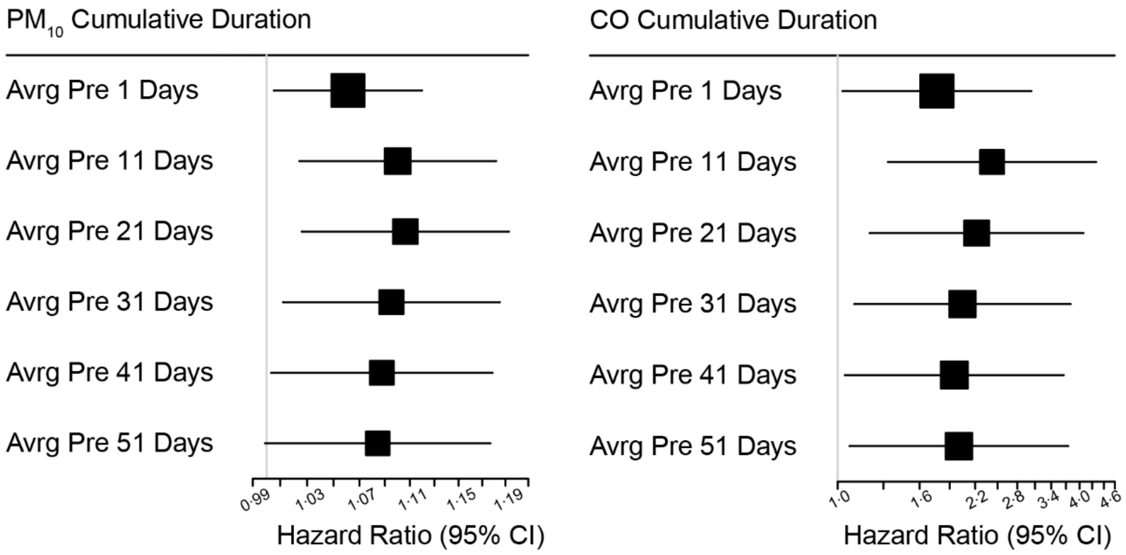

PM= particulate matter, CO = carbon monoxide, Avrg Pre= cumulative average exposure of preoperative period from the surgical diagnosis. These models were adjusted by age, sex, IDH mutation status, and MGMT promoter methylation status.

Fig. C.8 Postoperative cumulative average Cox hazard model of the Severance GBM cohort.

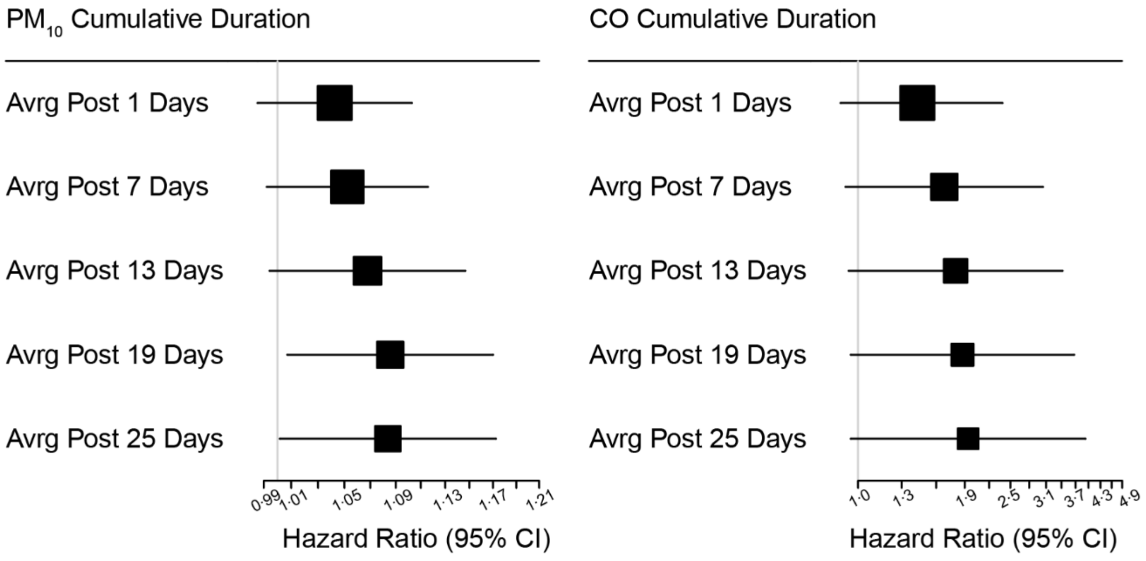

PM= particulate matter, CO = carbon monoxide, Avrg Post= cumulative average exposure after the surgical diagnosis in the Severance Hospital. These models were adjusted by age, sex, IDH mutation status, and MGMT promoter methylation status.

Fig. C.9 Short-Term Preoperative Exposure of Severance Cohort of GBM.

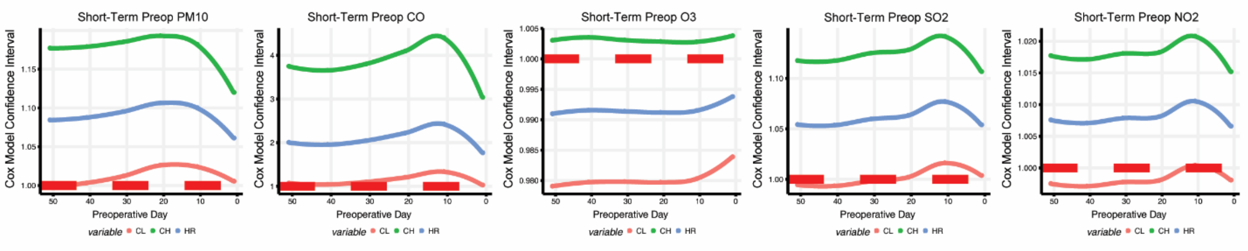

Preoperative short-term single pollutant Cox Hazard models of cumulative average of PM_{10}, CO, O_3, SO_2, and NO_2 are adjusted by age, sex, the IDH mutation status, the MGMT methylation status from preoperative 51st day to the 1st day. PM_{10}, CO, SO_2, and NO_2 show an interval in this short-term window.

Fig. C.10 Short-Term Postoperative Exposure of Severance Cohort of GBM.

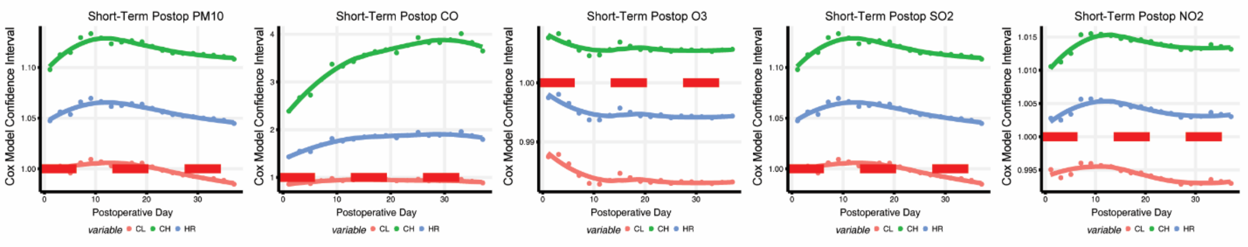

Short-term postoperative single pollutant Cox Hazard models of cumulative average of PM_{10}, CO, O_3, SO_2, and NO_2 are adjusted by age, sex, the IDH mutation status, the MGMT methylation status from postoperative 1st day to the 37th day. PM_{10} and SO_2 show a short interval of statistical significance.

SECTION D: Protocol-based validation in the Severance cohort.

APPENDIX TABLES

Table D.1 Demographics of Severance cohort study subjects and events, according to the exposure groups against PM_{10} (N=398).

Quintile	Q1	Q2	Q3	Q4	Q5
Range ($\mu g/m^3$)	20.8 - 35.4	35.5 - 42.7	42.8 - 51.7	51.8 - 60.5	60.6 - 105.8
Number of subjects	80	79	80	79	80
Mortality Events [a]	49	55	42	50	63
Age - yr	57.9 ± 15.4	56.7 ± 13.6	56 ± 12.7	57.7 ± 12.4	55.5 ± 12.9
Gender Male - %	68.75	55.70	60.00	56.96	62.50
IDH mutation - %	10.00	10.13	13.75	5.06	7.50
MGMT promoter methylation - %	42.50	31.65	46.25	32.91	40.00
Surgery-radiation sequence – Adjuvant %	88.75	82.28	86.25	82.28	81.25
Radiation type – Beam %	93.75	87.34	96.25	89.87	86.25
Chemotherapy status - Yes %	80.00	82.28	77.50	86.08	82.50

Plus–minus in the age indicates means ± standard deviation.
[a]Mortality events include all the causes of death.

Table D.2 Demographics of Severance cohort study subjects and events, according to the exposure groups against CO (N=396).

Quintile	Q1	Q2	Q3	Q4	Q5
Range (*ppm*)	0.2 - 0.4	0.4 - 0.5	0.5 - 0.6	0.6 - 0.7	0.7 - 1.2
Number of subjects	80	79	79	79	79
Mortality Events [a]	47	46	49	57	62
Age - yr	56.5 ± 12.7	57.5 ± 15	53.7 ± 13.8	57.6 ± 11.7	57.5 ± 14.1
Male - %	66.25	60.76	56.96	63.29	56.96
IDH mutation - %	8.75	11.39	13.92	5.06	7.59
MGMT promoter methylation - %	45	40.51	31.65	36.71	40.51
Surgery-radiation sequence – Adjuvant %	85	84.81	87.34	82.28	82.28
Radiation type – Beam %	91.25	91.14	93.67	88.61	88.61
Chemotherapy status - Yes %	87.5	77.22	84.81	78.48	86.08

Plus–minus in the age indicates means ± standard deviation.
[a]Mortality events include all the causes of death.

APPENDIX FIGURES

Fig. D.1 Survival curve of the perioperative exposure of the Severance cohort.

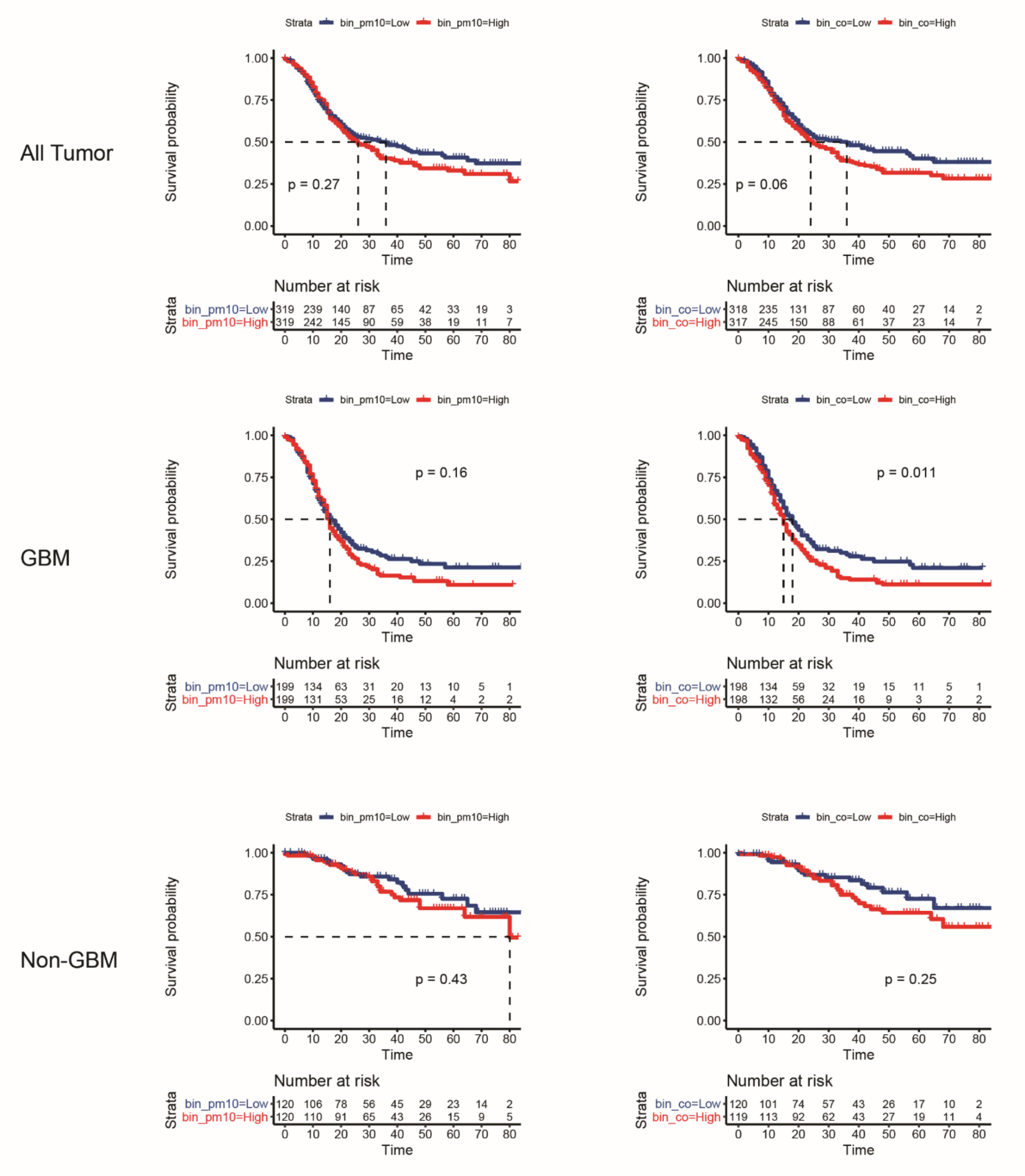

The overall survival result of PM_{10} and carbon monoxide (Median survival of GBM patients with PM_{10}= 16 months each, GBM with CO = 15 vs 18 months).

Fig. D.2 Distance Analysis of PM_{10} in the Severance Cohort.

Model Names	Beta	HR	N
Main Model with 5 km limit	0.009	1.009	373
Main Model with 10 km limit	0.009	1.009	400
Main Model with 50 km limit	0.008	1.008	420

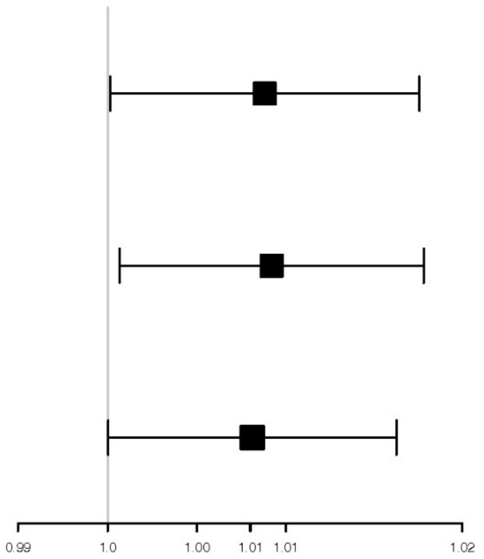

Single pollutant Cox Hazard model adjusted by age, sex, IDH mutation status, and MGMT methylation status.

Fig. D.3 Distance Analysis of CO in the Severance Cohort.

Model Names	Beta	HR	N
Main Model with 5 km limit	0.631	1.88	373
Main Model with 10 km limit	0.707	2.027	400
Main Model with 50 km limit	0.677	1.968	420

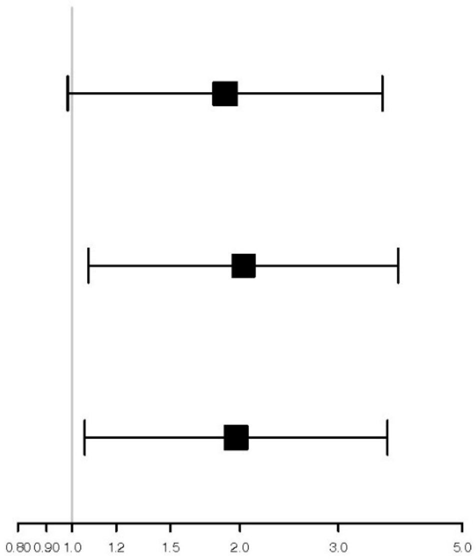

Single pollutant Cox Hazard model adjusted by age, sex, IDH mutation status, and MGMT methylation status.

Fig. D.4 Distance Analysis of O_3 in the Severance Cohort.

Model Names	Beta	HR	N
Main Model with 5 km limit	−0.008	0.992	373
Main Model with 10 km limit	−0.009	0.991	400
Main Model with 50 km limit	−0.007	0.993	420

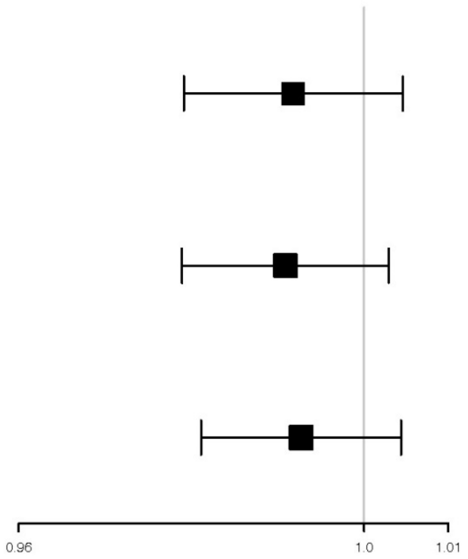

Single pollutant Cox Hazard model adjusted by age, sex, IDH mutation status, and MGMT methylation status.

Fig. D.5 Distance Analysis of SO_2 in the Severance Cohort.

Model Names	Beta	HR	N
Main Model with 5 km limit	0.063	1.065	373
Main Model with 10 km limit	0.068	1.07	400
Main Model with 50 km limit	0.056	1.057	420

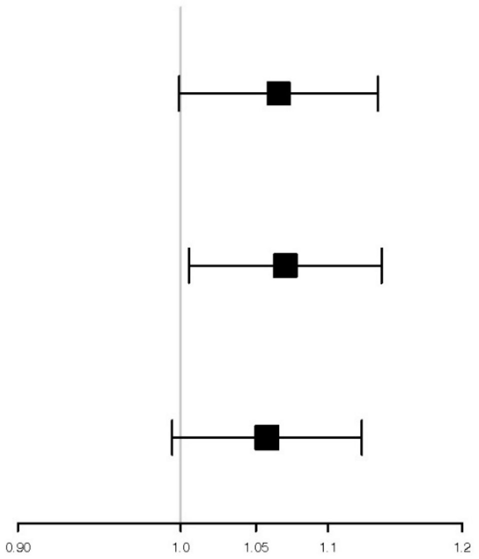

Single pollutant Cox Hazard model adjusted by age, sex, IDH mutation status, and MGMT methylation status.

Fig. D.6 Distance Analysis of NO_2 in the Severance Cohort.

Model Names	Beta	HR	N
Main Model with 5 km limit	0.008	1.008	373
Main Model with 10 km limit	0.008	1.008	400
Main Model with 50 km limit	0.007	1.007	420

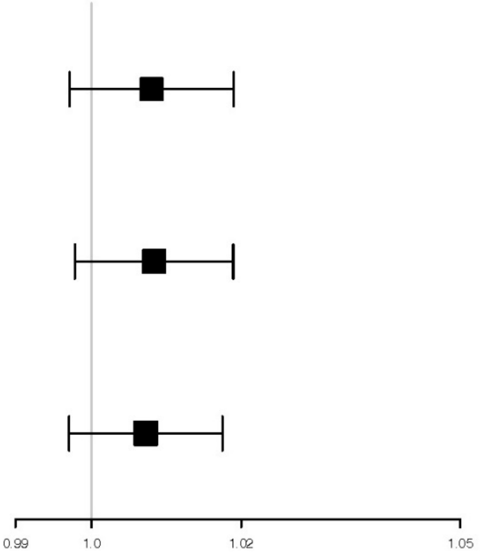

Single pollutant Cox Hazard model adjusted by age, sex, IDH mutation status, and MGMT methylation status.

SECTION E: Protocol-based validation in the SEER cohort.

APPENDIX TABLES

Table E.1 Demographics of SEER GBM patients by quintile of PM_{10} (N=10 621).

Quintile	Q1	Q2	Q3	Q4	Q5
Range ($\mu g/m^3$)	4.8 - 17.5	17.6 – 21.2	21.24 - 25.4	25.48 - 32.2	32.25 - 99.0
Number of subjects	2 134	2 124	2 149	2 125	2 089
Mortality events [a]	1 856	1 777	1 911	1 936	1 964
Age - yr	63.9 ± 13.6	63.6 ± 14.1	64.3 ± 13.7	64.1 ± 14.2	64.2 ± 14.1
Race					
American Indian/Alaska Native	0.23	0.28	0.00	0.09	0.19
Asian or Pacific Islander	12.79	9.04	7.40	5.88	4.26
Black	6.28	5.89	4.89	5.41	4.40
Hispanic (All Races)	13.92	17.37	16.85	18.87	17.23
White	66.73	67.28	70.45	69.69	73.82
Other	0.05	0.14	0.42	0.05	0.10
Surgery %	65.90	66.86	64.94	64.98	64.13
Chemotherapy %	59.93	58.57	56.44	49.60	41.41
Beam Radiation Therapy %	71.09	66.57	64.08	62.82	62.18
Education (%) [b]					
Below high School	13.55	16.64	17.01	18.12	18.33
High school to associate degree	47.57	46.95	48.13	48.67	52.53
College or higher education	38.88	36.41	34.87	33.22	29.14
Income [b]					
<$50,000	8.01	8.24	8.56	8.09	3.93
$50,000-79,999	58.48	65.77	76.13	82.12	91.34
≥$80,000	33.51	25.99	15.31	9.79	4.74
Married (%)	62.18	60.40	60.82	60.09	59.84
Moved last year % [b]					
No movement	86.37	86.47	86.22	86.21	85.65
Same county	8.18	8.95	9.37	9.57	9.70
Same state, different county	2.18	1.65	1.64	1.53	1.47
Different state	2.24	2.00	1.89	1.86	2.42
From Outside US	1.03	0.94	0.88	0.83	0.75

[a] Mortality events include all the causes of death.
[b] Education levels, income levels, and moving rates are calculated from the American Community Survey Tables: 2011- 2015 (5-Year Estimates).

Table E.2 Demographics of SEER GBM patients by the level of CO (N=10 621).

Quintile	Q1	Q2	Q3	Q4	Q5
Range (*ppm*)	0 - 0.345	0.346 - 0.458	0.459 - 0.602	0.603 - 0.806	0.807 - 2.361
Number of subjects	2 129	2 121	2 125	2 130	2 116
Mortality events [a]	1 829	1 808	1 875	1 910	2 022
Age - yr	64.6 ± 13.4	63.9 ± 13.8	63.6 ± 14.3	64.2 ± 14.1	63.8 ± 14.0
Race					
American Indian/Alaska Native	0.23	0.14	0.09	0.14	0.19
Asian or Pacific Islander	9.35	7.21	7.67	8.78	6.43
Black	4.13	5.80	5.46	5.68	5.81
Hispanic (All Races)	14.28	16.97	17.13	18.83	17.01
White	71.91	69.64	69.46	66.38	70.51
Other	0.09	0.24	0.19	0.19	0.05
Surgery %	71.40	72.80	72.52	72.39	70.18
Chemotherapy %	61.34	59.55	54.35	49.67	41.26
Beam Radiation Therapy %	65.90	66.86	64.94	64.98	64.13
Education (%) [b]					
Below high School	15.00	16.97	16.60	17.37	17.68
High school to associate degree	35.14	34.57	34.88	34.19	33.86
College or higher education	49.87	48.46	48.53	48.44	48.46
Income [b]					
<$50,000	14.28	4.43	6.73	5.26	6.19
$50,000-79,999	58.85	78.60	74.21	80.61	81.29
≥$80,000	26.87	16.97	19.06	14.13	12.52
Married (%)	62.00	60.07	61.93	58.31	61.06
Moved last year % [b]					
Same house	85.68	86.30	86.32	86.31	86.33
Same county	9.20	9.11	9.00	9.17	9.28
Same state, different county	2.42	2.07	2.07	1.95	1.89
Different state	1.80	1.65	1.72	1.69	1.61
From Outside US	0.90	0.87	0.89	0.88	0.89

Plus–minus in the age indicates means ± standard deviation.
[a] Mortality events include all the causes of death.
[b] Education levels, income levels, and moving rates are calculated from the American Community Survey Tables: 2011- 2015 (5-Year Estimates).

Table E.3 Causes of death of the SEER GBM patients in the perioperative single-pollutant models.

	Number of Events	Hazard Ratio (95% CI)
PM_{10} (10 µg/m^3)		Overall exposure
Overall cause	9 444	**1.044 (1.025 - 1.063)**
Brain cause	8 199	**1.049 (1.029 - 1.07)**
Cardiovascular causes	204	**1.127 (1.01 - 1.258)**
Pulmonary causes	63	1.041 (0.838 - 1.294)
CO (1 ppm)		Overall exposure
Overall cause	9 444	**1.075 (1.006 - 1.148)**
Brain cause	8 199	1.046 (0.975 - 1.123)
Cardiovascular causes	204	**1.746 (1.169 - 2.608)**
Pulmonary causes	63	0.959 (0.426 - 2.158)
Ozone (1 ppb)		Overall exposure
Overall cause	9 444	**1.004 (1.002 - 1.006)**
Brain cause	8 199	**1.004 (1.002 - 1.007)**
Cardiovascular causes	204	0.988 (0.974 - 1.002)
Pulmonary causes	63	**1.029 (1.003 - 1.056)**
SO_2 (1 ppb)		Overall
Overall cause	9 444	0.99 (0.983 - 0.998)
Brain cause	8 199	0.983 (0.975 - 0.992)
Cardiovascular causes	204	0.954 (0.898 - 1.014)
Pulmonary causes	63	0.967 (0.875 - 1.069)
NO_2 (1 ppb)		Overall exposure
Overall cause	9 444	0.998 (0.996 – 0.999)
Brain cause	8 199	0.998 (0.996 – 1.000)
Cardiovascular causes	204	**1.016 (1.002 - 1.029)**
Pulmonary causes	63	0.987 (0.962 - 1.013)
$PM_{2.5}$ (10 µg/m^3)		Overall exposure
Overall cause	3 020 [a]	1.025 (0.97 - 1.083)
Brain cause	2 647 [a]	1.03 (0.971 - 1.092)
Cardiovascular causes	52 [a]	0.722 (0.456 - 1.144)
Pulmonary causes	17 [a]	1.422 (0.694 - 2.918)
$PM_{2.5\text{-}10}$ (10 µg/m^3)		Overall exposure
Overall cause	3 020 [a]	0.999 (0.964 - 1.035)
Brain cause	2 647 [a]	1.003 (0.966 - 1.042)
Cardiovascular causes	52 [a]	**1.327 (1.058 - 1.665)**
Pulmonary causes	17 [a]	0.661 (0.393 - 1.113)

All estimates were adjusted for age, sex, race, radiotherapy methods, surgical sequence, and the status of chemotherapy.

[a] $PM_{2.5}$ data were sparse than that of PM_{10} and other pollutants.

Table E.4 Cox hazard model of single-pollutant by the subgroup of GBM in the SEER cohort.

		**PM_{10} (10 µg/m^3)**		**CO (ppm)**	
Characteristics	N	Estimates	P value	Estimates	P value
Age					
20-55	2 788	**1.048 (1.009 - 1.088)**	0.015	1.119 (0.979 - 1.28)	0.10
56-65	2 778	**1.078 (1.04 - 1.118)**	**<0.0001**	1.038 (0.909 - 1.185)	0.58
66-75	2 610	**1.055 (1.018 - 1.094)**	0.0035	1.104 (0.978 - 1.246)	0.11
76-98	2 445	1.024 (0.988 - 1.061)	0.18	**1.184 (1.04 - 1.348)**	0.010
Sex					
Female	4 366	**1.077 (1.046 - 1.108)**	**<0.0001**	**1.144 (1.033 - 1.267)**	**0.0097**
Male	6 255	**1.036 (1.013 - 1.061)**	**0.0026**	**1.117 (1.028 - 1.213)**	**0.0089**
Radiation surgery sequence					
None	4 903	**1.004 (1.001 - 1.006)**	**0.0054**	**1.11 (1.013 - 1.215)**	**0.024**
other	126	1.01 (0.991 - 1.029)	0.30	1.343 (0.71 - 2.541)	0.36
Adjuvant RTx	5 592	**1.007 (1.004 - 1.009)**	**<0.0001**	**1.108 (1.011 - 1.214)**	**0.028**
Chemotherapy					
No/Unknown	4 966	**1.004 (1.001 - 1.006)**	**0.0047**	**1.099 (1.009 - 1.196)**	**0.029**
Yes	5 655	**1.006 (1.003 - 1.009)**	**<0.0001**	**1.12 (1.016 - 1.235)**	**0.022**
Radiotherapy					
Beam	6 970	**1.005 (1.002 - 1.007)**	**0.00015**	1.047 (0.966 - 1.136)	0.26
no beam	3 651	**1.004 (1.001 - 1.007)**	**0.0065**	**1.192 (1.074 - 1.322)**	**0.00096**

All subgroups of these single-pollutant models are adjusted by age, sex, race, radiation method, radiation surgery sequence, chemotherapy status, and race.

Table E.5 Cox hazard model of single-pollutant by the location in the SEER cohort.

		PM_{10} (10 μg/m^3)		CO (ppm)	
Residential location	N	Estimates	P value	Estimates	P value
California	7 758	**1.005 (1.003 - 1.007)**	**<0.0001**	**1.136 (1.058 - 1.219)**	**0.00045**
Not California	2 863	1.004 (0.999 - 1.009)	0.12	1.036 (0.886 - 1.211)	0.65

The single-pollutant models are adjusted by age, sex, race, radiation method, radiation surgery sequence, chemotherapy status, and race.

Table E.6 Cox hazard model of single-pollutant by the year of diagnosis in the SEER cohort.

		PM_{10} (10 μg/m^3)		CO (ppm)	
Year of diagnosis	N	Estimates	P value	Estimates	P value
2000	614	1.002 (0.995 - 1.009)	0.59	1.076 (0.893 - 1.297)	0.44
2001	613	**1.009 (1.001 - 1.018)**	0.035	0.966 (0.783 - 1.192)	0.75
2002	602	0.994 (0.986 - 1.002)	0.14	0.883 (0.696 - 1.119)	0.3
2003	670	1.004 (0.997 - 1.012)	0.23	1.051 (0.833 - 1.325)	0.68
2004	658	**1.01 (1.001 - 1.019)**	0.023	1.016 (0.765 - 1.35)	0.91
2005	733	1.007 (0.997 - 1.016)	0.18	1.245 (0.984 - 1.575)	0.068
2006	639	0.998 (0.991 - 1.006)	0.70	1.092 (0.797 - 1.495)	0.58
2007	716	1.004 (0.999 - 1.009)	0.091	0.747 (0.48 - 1.163)	0.2
2008	637	1.005 (0.998 - 1.012)	0.13	0.801 (0.526 - 1.221)	0.3
2009	665	**1.008 (1 - 1.015)**	0.041	1.235 (0.843 - 1.808)	0.28
2010	675	0.994 (0.985 - 1.003)	0.16	0.84 (0.505 - 1.397)	0.5
2011	685	1.003 (0.995 - 1.012)	0.43	1.074 (0.76 - 1.519)	0.68
2012	709	**1.011 (1.001 - 1.021)**	0.032	1.204 (0.763 - 1.899)	0.42
2013	669	0.996 (0.987 - 1.005)	0.36	0.758 (0.486 - 1.183)	0.22
2014	648	**1.014 (1.004 - 1.025)**	0.0080	0.699 (0.428 - 1.14)	0.15
2015	688	1.014 (0.999 - 1.03)	0.060	0.826 (0.39 - 1.748)	0.62

The single-pollutant models are adjusted by age, sex, race, radiation method, radiation surgery sequence, chemotherapy status, and race.

Table E.7 Cox hazard model of single-pollutant by the month of diagnosis in the SEER cohort.

		PM_{10} (10 μg/m^3)		CO (ppm)	
Month of diagnosis	N	Estimates	P value	Estimates	P value
January	950	**1.009 (1.002 - 1.015)**	0.011	**1.328 (1.11 - 1.589)**	0.0019
February	778	1.003 (0.993 - 1.013)	0.57	1.189 (0.931 - 1.518)	0.16
March	937	1.004 (0.997 - 1.012)	0.23	1.18 (0.882 - 1.581)	0.26
April	862	1.002 (0.995 - 1.01)	0.53	1.229 (0.879 - 1.719)	0.22
May	880	1.006 (0.998 - 1.014)	0.12	1.223 (0.858 - 1.742)	0.26
June	897	0.999 (0.992 - 1.006)	0.79	1.166 (0.813 - 1.672)	0.40
July	936	**1.007 (1.001 - 1.013)**	0.029	1.117 (0.785 - 1.589)	0.53
August	878	**1.007 (1.001 - 1.013)**	0.031	1.177 (0.825 - 1.678)	0.36
September	814	1.001 (0.994 - 1.007)	0.78	0.798 (0.552 - 1.155)	0.23
October	909	**1.008 (1.002 - 1.013)**	0.0034	1.24 (0.935 - 1.645)	0.13
November	882	**1.007 (1.002 - 1.012)**	0.010	**1.283 (1.007 - 1.634)**	0.043
December	898	**1.009 (1.003 - 1.015)**	0.0043	1.17 (0.979 - 1.398)	0.084

The single-pollutant models are adjusted by age, sex, race, radiation method, radiation surgery sequence, chemotherapy status, and race.

APPENDIX FIGURES

Fig. E.1 Sensitivity Analysis of PM_{10} in the SEER Cohort.

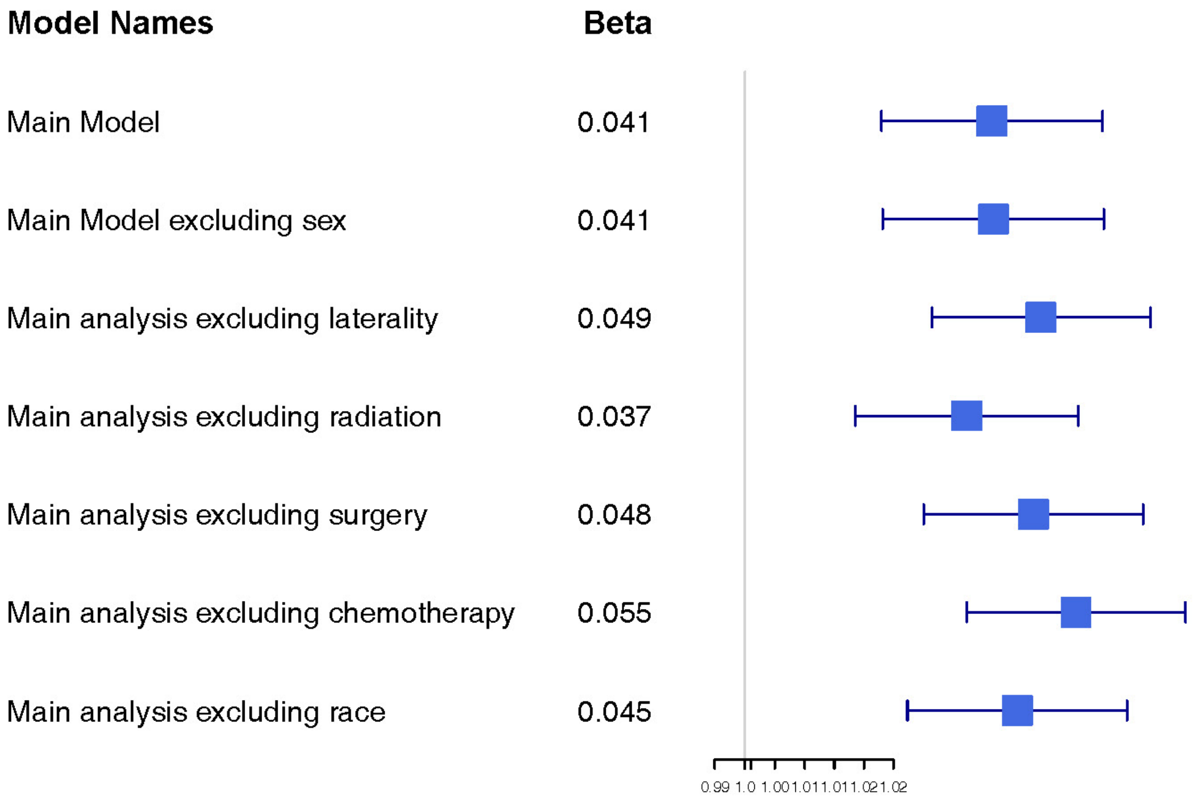

The main model of the single-pollutant was adjusted for age, sex, laterality of tumor, radiation type, surgery status, chemotherapy status, and race. The model was constructed with the patients within the 10 km from the air monitoring stations (N= 10621).

Fig. E.2 Sensitivity Analysis of CO in the SEER Cohort.

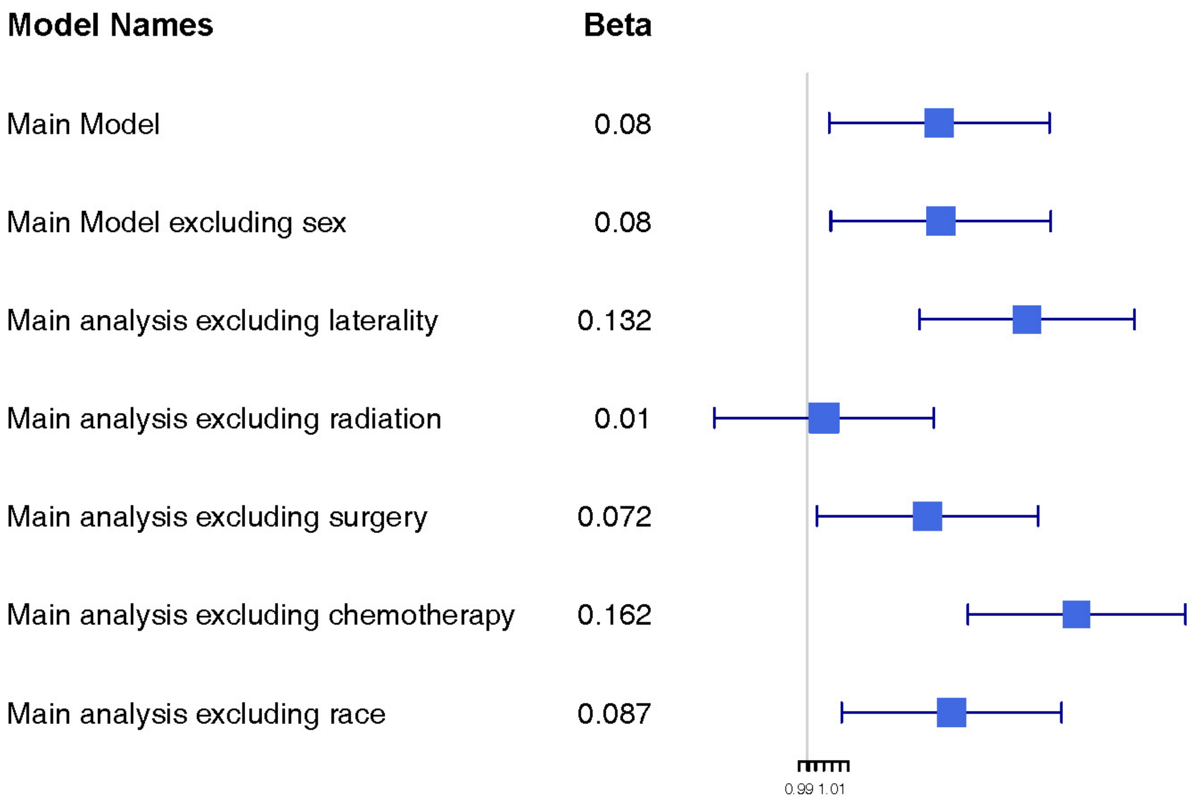

The main model of the single-pollutant was adjusted for age, sex, laterality of tumor, radiation type, surgery status, chemotherapy status, and race. The model was constructed with the patients within the 10 km from the air monitoring stations (N= 10621).

Fig. E.3 Sensitivity Analysis of O_3 in the SEER Cohort.

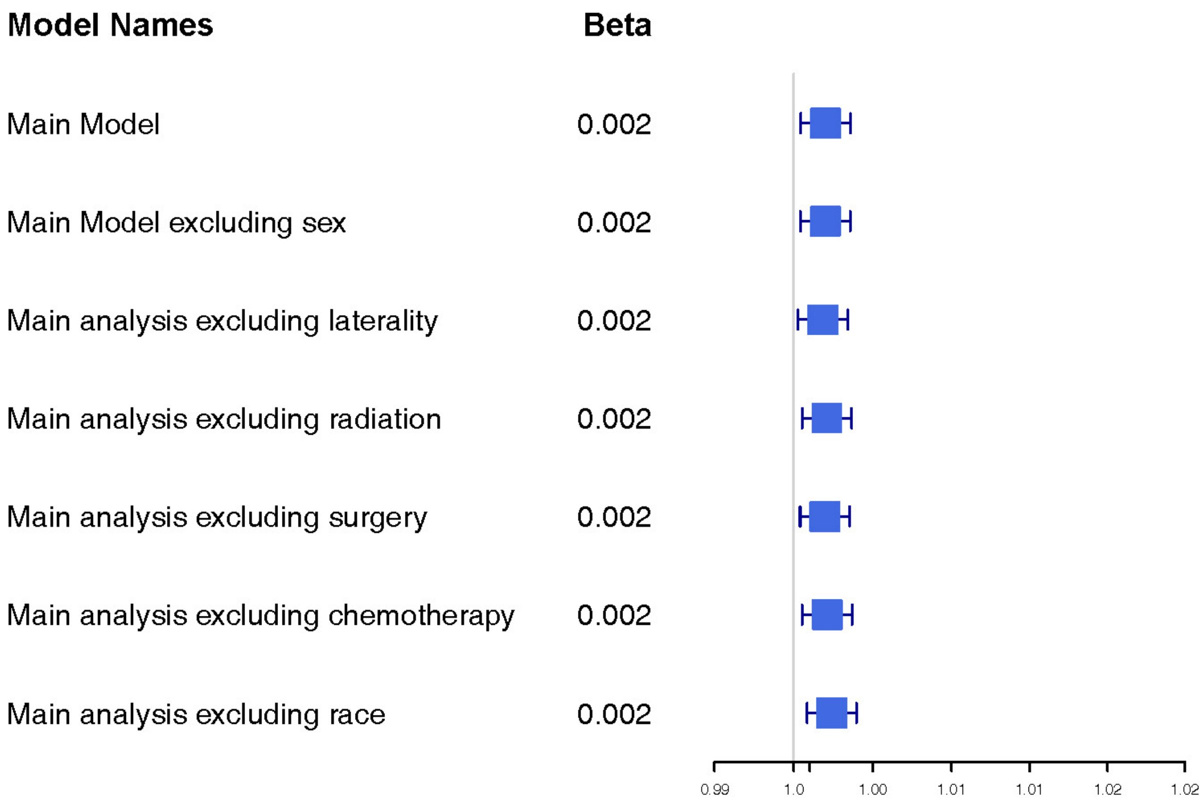

The main model of the single-pollutant was adjusted for age, sex, laterality of tumor, radiation type, surgery status, chemotherapy status, and race. The model was constructed with the patients within the 10 km from the air monitoring stations (N= 10621).

Fig. E.4 Sensitivity Analysis of SO_2 in the SEER Cohort.

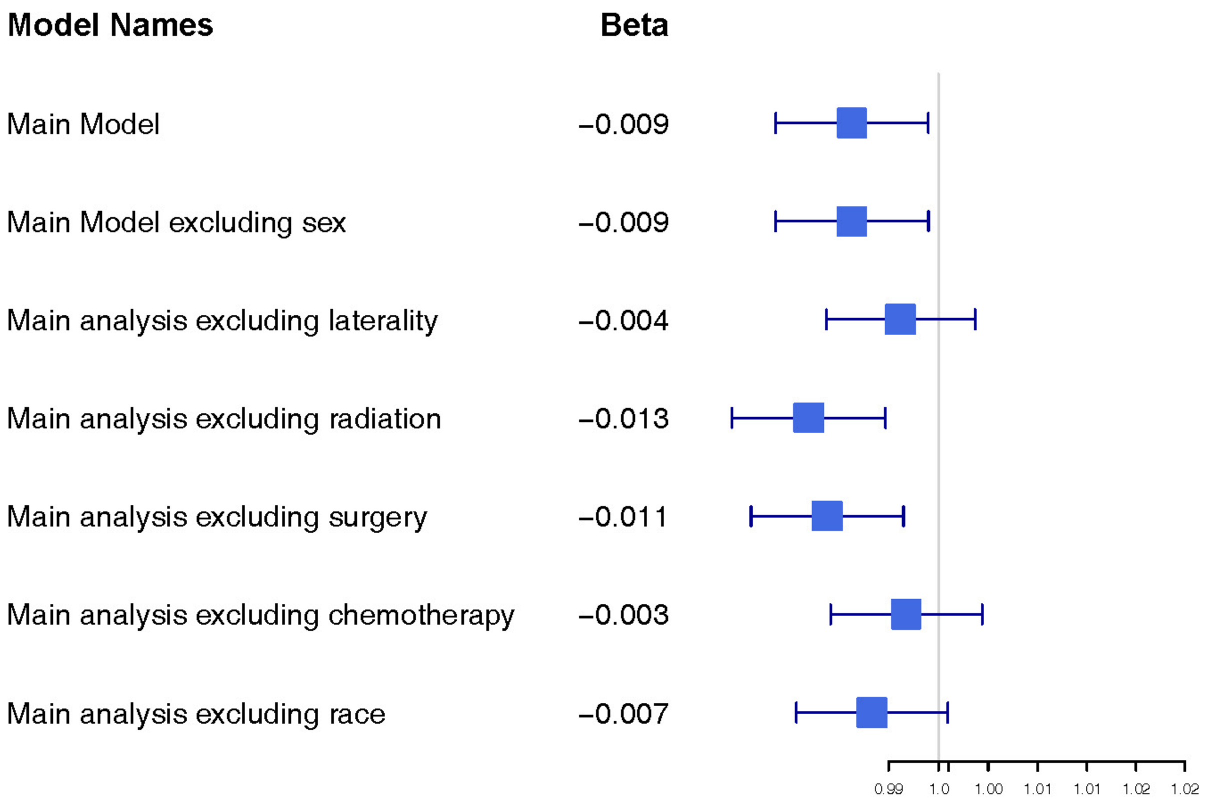

The main model of the single-pollutant was adjusted for age, sex, laterality of tumor, radiation type, surgery status, chemotherapy status, and race. The model was constructed with the patients within the 10 km from the air monitoring stations (N= 10621).

Fig. E.5 Sensitivity Analysis of NO_2 in the SEER Cohort.

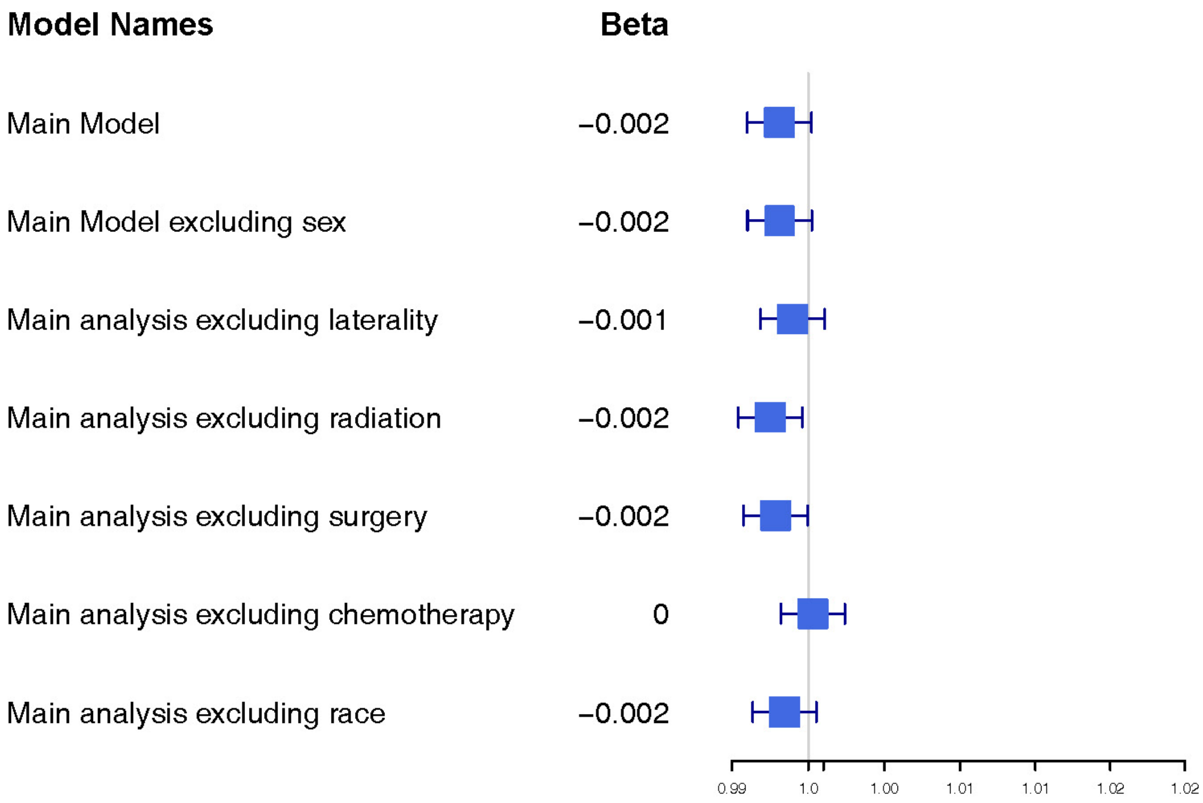

The main model of the single-pollutant was adjusted for age, sex, laterality of tumor, radiation type, surgery status, chemotherapy status, and race. The model was constructed with the patients within the 10 km from the air monitoring stations (N= 10621).

Fig. E.6 Distance Analysis of PM_{10} in the SEER Cohort.

Model Names	Beta	HR	N
Main Model with 10 km limit	0.043	1.044	10,621
Main Model with 50 km limit	0.012	1.012	28,849
Main Model with 100 km limit	0.012	1.012	35,490
Main Model with 200 km limit	0.011	1.011	40,297
Main Model with 500 km limit	0.011	1.011	42,293

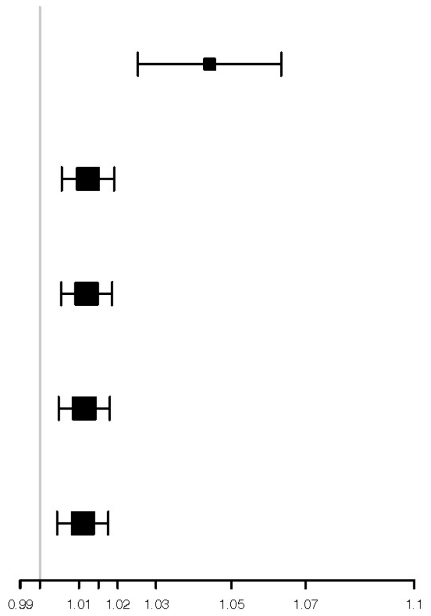

Cox Hazard Ratio of PM_{10} adjusted by age, sex, race, surgery, radiation, laterality, and chemotherapy.

Fig. E.7 Distance Analysis of CO in the SEER Cohort.

Model Names	Beta	HR	N
Main Model with 10 km limit	0.072	1.075	10,621
Main Model with 50 km limit	0.034	1.035	28,849
Main Model with 100 km limit	0.017	1.017	35,490
Main Model with 200 km limit	0.017	1.017	40,297
Main Model with 500 km limit	0.015	1.015	42,293

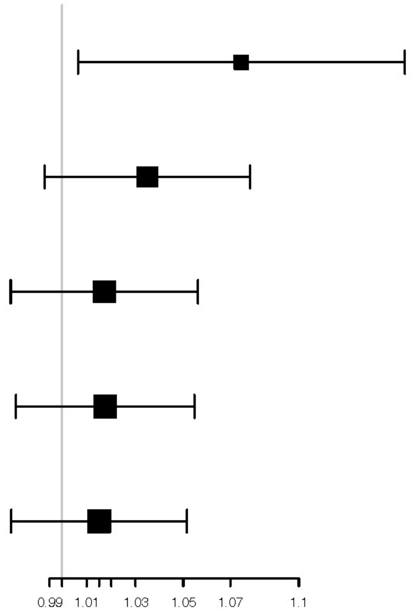

Cox Hazard Ratio of CO adjusted by age, sex, race, surgery, radiation, laterality, and chemotherapy.

Fig. E.8 Distance Analysis of O_3 in the SEER Cohort.

Model Names	Beta	HR	N
Main Model with 10 km limit	0.004	1.004	10,621
Main Model with 50 km limit	0.002	1.002	28,849
Main Model with 100 km limit	0.002	1.002	35,490
Main Model with 200 km limit	0.002	1.002	40,297
Main Model with 500 km limit	0.002	1.002	42,293

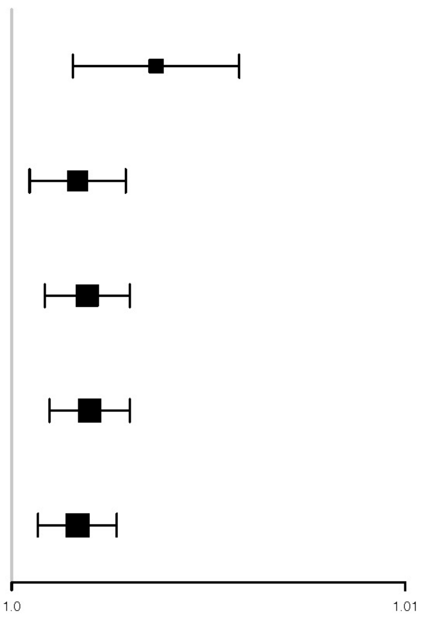

Cox Hazard Ratio of O_3 adjusted by age, sex, race, surgery, radiation, laterality, and chemotherapy.

Fig. E.9 Distance Analysis of SO_2 in the SEER Cohort.

Model Names	Beta	HR	N
Main Model with 10 km limit	−0.01	0.99	10,621
Main Model with 50 km limit	−0.006	0.994	28,849
Main Model with 100 km limit	−0.006	0.994	35,490
Main Model with 200 km limit	−0.006	0.994	40,297
Main Model with 500 km limit	−0.005	0.995	42,293

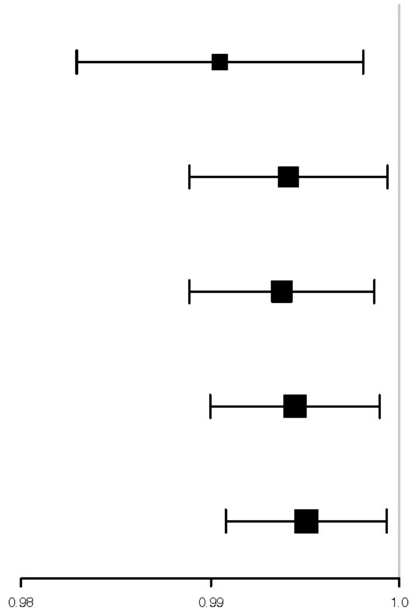

Cox Hazard Ratio of SO_2 adjusted by age, sex, race, surgery, radiation, laterality, and chemotherapy.

Fig. E.10 Distance Analysis of NO_2 in the SEER Cohort.

Model Names	Beta	HR	N
Main Model with 10 km limit	−0.002	0.998	10,621
Main Model with 50 km limit	−0.003	0.997	28,849
Main Model with 100 km limit	−0.003	0.997	35,490
Main Model with 200 km limit	−0.003	0.997	40,297
Main Model with 500 km limit	−0.003	0.997	42,293

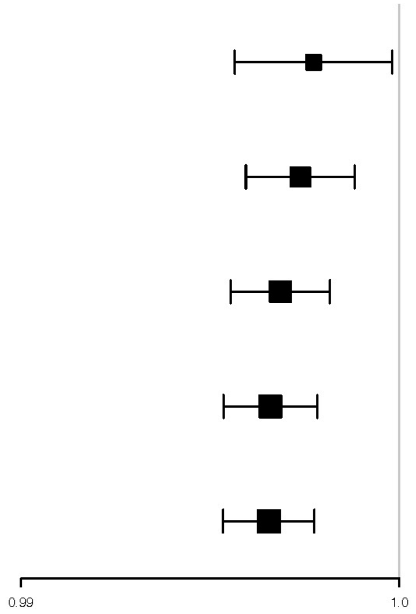

Cox Hazard Ratio of NO_2 adjusted by age, sex, race, surgery, radiation, laterality, and chemotherapy.

Fig. E.11 Distribution of perioperative exposure to patients in the SEER cohort.

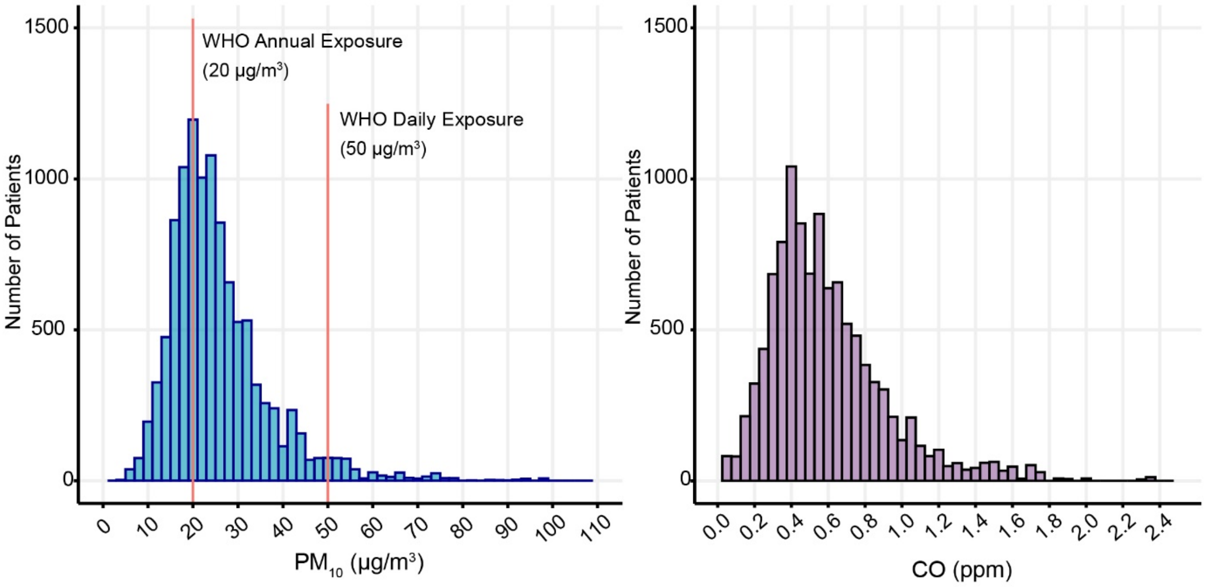

Perioperative exposure: The average cumulative exposure at the month of diagnosis, **WHO:** World health organization, **PM_{10}:** Particulate matter with an aerodynamic diameter less than 10 μm, **CO:** Carbon monoxide, **ppm:** Parts per million

Fig. E.12 The survival curve of the SEER database.

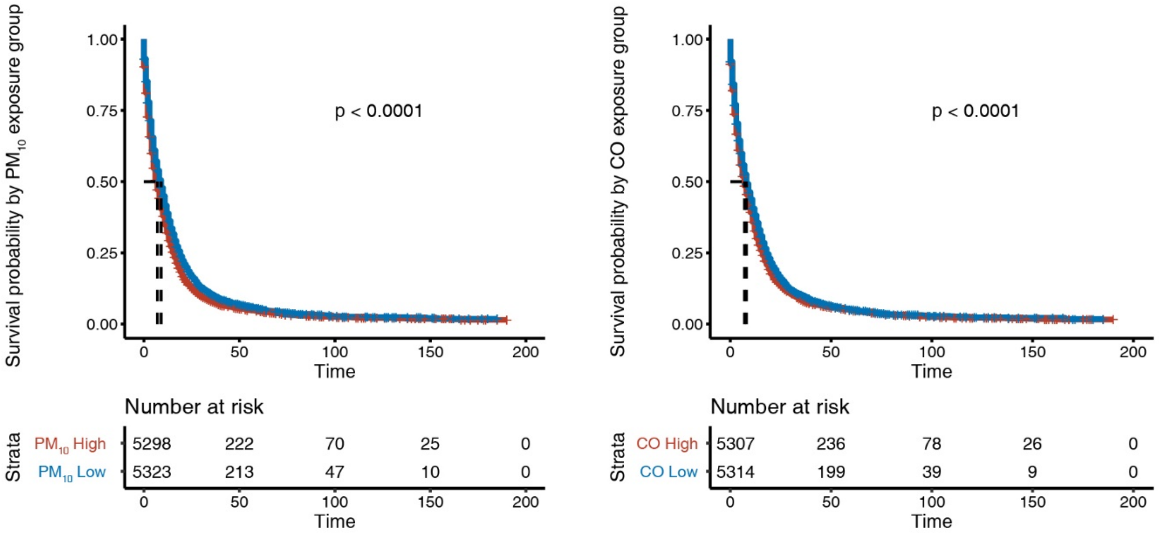

The survival difference was two months for PM_{10} and CO (The median survival 7 vs 9 months).

PM_{10}: Particulate matter with an aerodynamic diameter less than 10 μm, **CO:** Carbon monoxide

APPENDIX REFERENCES

1 Balss J, Meyer J, Mueller W, Korshunov A, Hartmann C, von Deimling A. Analysis of the IDH1 codon 132 mutation in brain tumors. *Acta Neuropathol* 2008; **116**: 597-602.

2 Yan H, Parsons DW, Jin G, et al. IDH1 and IDH2 mutations in gliomas. *N Engl J Med* 2009; **360**: 765-73.

3 Louis DN, Perry A, Reifenberger G, et al. The 2016 World Health Organization Classification of Tumors of the Central Nervous System: a summary. *Acta Neuropathol* 2016; **131**: 803-20.

4 National Cancer Institute D, Surveillance Research Program. SEER*Stat Database: Incidence - SEER 9 Regs Research Data, Nov 2018 Sub (1973-2015) Linked To County Attributes - Total U.S., 1969-2015 <Katrina/Rita Adjustment>. November 2017 submission.

5 Cromar KR, Duncan BN, Bartonova A, et al. Air Pollution Monitoring for Health Research and Patient Care. An Official American Thoracic Society Workshop Report. *Ann Am Thorac Soc* 2019; **16**: 1207-14.

6 Turner MC, Krewski D, Diver WR, et al. Ambient Air Pollution and Cancer Mortality in the Cancer Prevention Study II. *Environ Health Perspect* 2017; **125**: 087013.

7 Miller KA, Siscovick DS, Sheppard L, et al. Long-term exposure to air pollution and incidence of cardiovascular events in women. *N Engl J Med* 2007; **356**: 447-58.

8 Schoenfeld DA. Sample-size formula for the proportional-hazards regression model. *Biometrics* 1983; **39**: 499-503.
